# Mild-Temperature Synthesis of Gold Colloids with Unique Features Mediated by Polymers of Biomedical Interest

**DOI:** 10.1021/acsomega.5c01123

**Published:** 2025-05-22

**Authors:** Gabriela Borba Mondo, Caroline Arana da Silva Ribeiro, Alžběta Turnovská, Michaela Hrochová, Tomáš Etrych, Fernanda Dias da Silva, Cristiano Giacomelli, Fernando C. Giacomelli

**Affiliations:** a Centro de Ciências Naturais e Humanas, Universidade Federal do ABC, Santo André 09280-560, Brazil; b Institute of Macromolecular Chemistry, Czech Academy of Sciences, Prague 162 00, Czech Republic; c Departamento de Química, Centro de Ciências Naturais e Exatas, 28118 Universidade Federal de Santa Maria, Santa Maria 97105-900, Brazil

## Abstract

Well-defined gold nanoparticles (AuNPs) are accessible via simple synthetic methods, and their surface chemistry stands as a key factor in determining applications in the biomedical field. While macromolecules featuring amino groups are already known to successfully mediate the formation of stable gold colloids in one-pot, two-reactant, no workup reactions in aqueous media, we herein report the discovery that, under mild reaction temperatures, polymers of outstanding biomedical interest not only can play the simultaneous role of reducing and capping agent but also lead to particulate systems with unique features. From a library of samples that included branched polyethylenimine (BPEI), poly­(l-lysine) (PLL), bovine serum albumin (BSA), poly­(2-methyl-2-oxazoline) (PMeOx), poly­(*N*-(2-hydroxypropyl) methacrylamide) (PHPMA), and amine-functionalized poly­(*N*-(2-hydroxypropyl)­methacrylamide-*co*-*N*-(3-aminopropyl)­methacrylamide) P­(HPMA-*co*-APMA), we found that PHPMA end-functionalized with nitrile motifs generate spherical and stable AuNPs@PHPMA of very small size (diameter of ∼2.4 nm), as underlined by imaging experiments. Cell viability experiments indicated exceptionally good biocompatibility up to very high numerical particle concentrations as compared to the other systems. The reduced size imparted to the AuNPs@PHPMA outstanding catalytic properties (no induction time and high reaction rate constant for the hydrogenation of *p*-nitrophenol) and antimicrobial activity (total antibacterial activity against Escherichia coli and dose-dependent antibacterial activity against Staphylococcus aureus). The introduction of primary amine groups (13.4 mol %) of higher nucleophilicity known to work better for AuNP synthesis makes these unique features disappear, as evidenced for P­(HPMA-*co*-APMA). The other systems yielded 6–28 nm particles whose properties reflected both the size of the metallic core and chemical nature and conformation of the capping agent. These findings point to novel applications of PHPMA polymers worthy of further development, especially in light of their excellent water solubility and biocompatibility.

## Graphical abstract

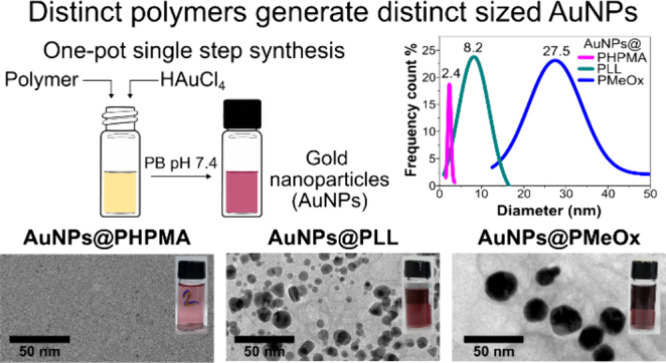

## Introduction

The surface chemistry of gold colloids is a key factor in determining their applications for the manufacturing of advanced materials, to which they impart unique characteristics, especially at the nanoscale. Here, the high surface-to-volume ratio generates strong adhesion forces that require the use of capping agents in order to overcome disordered agglomeration and sterically stabilize the particles. However, by employing capping agents, the interfacial activity is altered, directly affecting their features and behavior.^1^ Therefore, such systems must be properly designed from a holistic and integrative perspective, which certainly is a challenging endeavor because the physical and mechanistic inorganic/organic chemistry underlying the synthesis and the functioning of these materials are rather complex and remain the subject of investigation.

Yet, the fact that a vast field of opportunities is accessible via synthetic strategies as simple as a one-pot, two-reactant, no workup reaction in aqueous media is surely amazing. On one hand, the state-of-the-art macromolecular engineering techniques allow for the synthesis of a myriad of polymers with controlled chemical composition, functionality, and topography at different levels of sophistication and complexity.^2^ On the other hand, hybrid organic–inorganic polymer-coated gold nanoparticles (AuNP@polymer) can be synthesized by reacting gold­(III) with polymers that simultaneously play the roles of reducing and stabilizing agents.

In this regard, recent advances have proven that the minimal requirement for polymers to promote the formation of well-defined gold colloids is the presence of electron-donor atoms at functional moieties that ultimately lead to spontaneous reactions. From a thermodynamic standpoint, this is possible whenever their reduction potential lies between that of the oxidation of Au(0) to Au­(I) and the reduction of Au­(III) to Au(0), as evidenced for low molecular weight amines,^3^ meaning that many systems are eligible for this purpose. Indeed, an array of opportunities spans from the fundamental knowledge that simple amines react with auric ions yielding metallic gold and an oxidized amine as a product in the first step. Then, the latter further prompts an oxidative polymerization process that generates a polymeric coating on the nanoparticle surface while gold cluster growth also takes place.^4^ Therefore, the fate of oxidized amines (radical species) known to this date^3–5^ means that a starlike structure consisting of several chains is possibly generated by intermolecular cross-linking around the metallic core when the reducing motifs are part of the polymer chains (similar to the *grafting to* technique). In addition, polymer chains may also simply chemisorb onto the surface of newly grown metallic particles following a typical Pearson’s hard–soft acid–base principle. Tethered to the surface, the polymeric shell dictates the dynamics of interaction between the particles themselves and with the surrounding environment and, as a consequence, also defines the field of application.

Within this framework, the right balance between redox potentials and bond formation involving nitrogen-containing (macro)­molecules and gold species has paved a very interesting way to new materials. The most popular are polymers with either primary, secondary, or tertiary amino groups. Early studies carried out in our laboratories^6,7^ and elsewhere^8^ established that AuNP formation at room temperature was dependent on the aqueous dissociation equilibrium involving amino groups and the Au­(III) speciation, pointing that amino groups would have to remain unprotonated to a quite significant degree with a minimum [N]/[Au] ratio required to obtain well-defined particles. Later, Mountrichas et al.^9^ discovered that the reaction temperature becomes a very important variable, especially under acidic conditions where only a very small fraction of amino groups remain unprotonated, reporting that the higher the temperature, the faster nanoparticle synthesis is and the more uniform the resulting objects are. Upon rising the reaction temperature, polymers featuring other nitrogen-based chemical functions such as amides and amino acids, either at the backbone or as pendant groups, were indeed found to serve the dual roles of reducing and capping agents.^7,10^ Since many of the macromolecules of utmost importance for advanced biomedical applications do fall into this category, there is renewed interest in synthesizing AuNPs with such systems at elevated temperature.

Inspired by these observations, we built a representative library of macromolecules that have shown promising potential for applications in biological systems, which comprises a branched polyethylenimine (BPEI), a poly­(l-lysine) (PLL) homopolymer, the bovine serum albumin (BSA) protein, a linear poly­(2-methyl-2-oxazoline) (PMeOx), a poly­(*N*-(2-hydroxypropyl)­methacrylamide) (PHPMA), and an amine-functionalized PHPMA-based poly­(*N*-(2-hydroxypropyl)­methacrylamide-*co*-*N*-(3-aminopropyl)­methacrylamide) copolymer P­(HPMA-*co*-APMA). BPEI and PLL have been used to build complexes with other biomolecules via electrostatic interactions specially designed for nonviral gene delivery, while BSA is a well-known protein. PMeOx belongs to the class of peptidomimetic polymers with growing significance in the medical industry^11,12^ particularly due to their excellent protein-repellent properties,^13^ which, together with PHPMA, contributes to overcoming some of the well-known issues related to poly­(ethylene oxide) technology.^11^ PHPMA-based copolymers are an interesting platform for biorelevant studies because their excellent water solubility and biocompatibility allow for the design of multifunctional systems incorporating therapeutic drugs, contrast agents, targeting moieties, and drug release triggers,^14,15^ possibly all-in-one. The set includes samples with all types of primary, secondary, and tertiary amino groups, primary and secondary amide groups, and derivatives of amino acids.

In the present study, we address fundamental aspects of the synthesis process at mild temperatures and subsequent characterization and performance of polymer- or protein-coated AuNPs in catalytic, cytotoxicity, and antimicrobial activity assays. The findings not only confirm the feasibility of direct synthesis by all of the selected macromolecules but also lead to discovery and novel methodology for manufacturing very small gold nanoparticles.

## Experimental Section

### Materials and Chemicals

Branched polyethylenimine (BPEI, average *M*_n_ 10,000 g·mol^–1^, *M*_w_/*M*_n_ = 2.5), BSA, poly­(l-lysine) (PLL, average *M*_n_ 17,000 g·mol^–1^, *M*_w_/*M*_n_ ≤ 1.4), hydroxy terminated poly­(2-methyl-2-oxazoline) (PMeOx, average *M*_n_ 5000 g·mol^–1^, *M*_w_/*M*_n_ < 1.3), chloroauric acid (HAuCl_4_·3H_2_O), sodium hydroxide (NaOH), sodium phosphate dibasic dodecahydrate (Na_2_HPO_4_·12H_2_O), and potassium phosphate monobasic (KH_2_PO_4_) were of the highest purity available from Sigma-Aldrich and used as received. 2,2′-Azobis­(isobutyronitrile) (AIBN), methacroyl chloride, 1-aminopropan-2-ol, 6-aminohexanoic acid, *N*-(3-(dimethylamino)­propyl)-*N*′-ethylcarbodiimide hydrochloride (EDC), carbon disulfide, ethanthiol, sodium hydride (60% dispersion in mineral oil), 5-hydroxy-2-pentanone, 2-thiazoline-2-thiol, 4,6-trinitrobenzene-1-sulfonic acid (TNBSA), *t*-butanol (*t*-BuOH), *N*,*N*-dimethylacetamide (DMA), and dimethyl sulfoxide (DMSO) were obtained from Merck (Czech Republic). Diethyl ether, acetone, and methanol (MeOH) were obtained from Lachner (Czech Republic), and *N*-(3-BOC-aminopropyl)­methacrylamide (APMA-Boc) was obtained from Polysciences, Inc. (USA). Azoinitiator 2,2′-azobis­(4-methoxy-2,4-dimethylvaleronitrile) (V-70) was obtained from Wako Pure Chemical Industries Ltd. (Japan). All the solvents used were of analytical grade.

### Synthesis of Monomers and Chain Transfer Agent

*N*-(2-Hydroxypropyl)­methacrylamide (HPMA) was synthesized via the reaction of methacryloyl chloride with 1-aminopropan-2-ol in dichloromethane (DCM) in the presence of sodium hydrogen carbonate as described in the literature.^16^ The trithiocarbonate chain transfer agent *S*-2-cyano-2-propyl-*S*′-ethyl trithiocarbonate (ethyl-TTc-AIBN) was synthesized as described by Ishitake et al.^17^

### Synthesis of the PHPMA and P­(HPMA-*co*-APMA) Polymers

Homopolymer PHPMA was prepared via the controlled radical reversible addition–fragmentation chain transfer (RAFT) polymerization of HPMA in the 85/15 (*v*/*v*) *t*-BuOH/DMA mixture, in the presence of V-70 as initiator and ethyl-TTc-AIBN as chain transfer agent (CTA). The monomer/CTA/initiator ratio was set at 140/1/0.5. The reaction was carried out as follows: HPMA (2.79 mmol, 0.40 g) was dissolved in *t*-BuOH (3.99 mL), while ethyl-TTc-AIBN (19.95 mmol, 5.40 mg) and V-70 (9.98 μmol, 4.05 mg) were dissolved in DMA (700 μL). Both solutions were mixed inside an ampule, bubbled for 10 min with argon, and sealed. After 20 h at 40 °C, the mixture was precipitated into acetone/diethyl ether (2/1), followed by filtering off and drying under vacuum of the precipitated polymer. Potentially cytotoxic TTc-end groups were removed by dissolving PHPMA (200.00 mg) and AIBN (40.00 mg) in DMA (1.60 mL), inserted into an ampule, bubbled with argon (10 min), and left at 80 °C for 3 h. The final PHPMA precursor was precipitated into acetone/diethyl ether (2/1), filtered, and dried under vacuum to a constant weight yielding 75%. GPC chromatograms recorded using a UV detector at 310 nm showing successful (98%+) potentially cytotoxic TTc-end group replacement by isobutyronitrile motifs are evidenced in Figure S1 (Supporting Information).

Polymer P­(HPMA-*co*-APMA) with amine groups alongside the chain was prepared analogously by RAFT polymerization of HPMA (2.79 mmol, 0.40 g) with APMA-Boc (0.49 mmol, 0.11 g) with V-70 (1.31 μmol, 4.05 mg) as initiator and ethyl-TTc-AIBN (2.63 μmol, 5.40 mg) as CTA. The reaction was carried out in 4.70 mL of *t*-BuOH/DMA (85/15) (*v*/*v*) at 40 °C for 20 h. The monomer/CTA/initiator ratio was set at 125/1/0.5, with the ratio of monomers HPMA/APMA-Boc 85/15. For the removal of TTc-end groups, the polymer (200.00 mg) was reacted with AIBN (40.00 mg) in DMA (1.6 mL) at 80 °C for 3 h. Polymer (125.00 mg) was boiled in Q-water (1.25 mL) at 150 °C for 1.5 h for the thermal deprotection (Boc group removal) of amine groups. Final polymer P­(HPMA-*co*-APMA) was obtained by lyophilization. The content of amine groups was determined using UV/vis spectroscopy, as described in detail below (see Methods/UV–Vis Spectroscopy sections). Size exclusion chromatography was used for the characterization of both PHPMA and P­(HPMA-*co*-APMA) polymers. Their detailed synthesis scheme is depicted in Scheme S1 (Supporting Information File), and their characterization is summarized in Table 1.

**Table 1:** Polymer- and Protein-Based Stabilizers Used in the AuNP Formation and Their Physical Properties: Number-Average Molar Mass (*M*_n_) and Molar Mass Dispersity (*Đ*_M_)­[Table-fn t1fn5]

**polymer**	*M*_**n**_ **(g·mol** ^**–1**^ **)**	***Đ*_M_** [Table-fn t1fn5]	**main characteristics of interest for AuNP synthesis**
BPEI	10,000^a^	2.5^a^	primary, secondary and tertiary nitrogen atoms (amines) at 1:2:1 ratio
BSA	66,463^b^	1^b^	583 amino acids; nitrogen atoms of diverse types in peptide bonds and as side chains; sulfur atoms also present.^25^
PLL	17,000^a^	≤1.4^a^	primary nitrogen atoms (amines) separated by butyl spacer from the backbone with a secondary nitrogen (amines) at 1:1 ratio
PMeOx	5,000^a^	<1.3^a^	tertiary nitrogen atoms (amides) per repeating unit at the backbone.
PHPMA	11,100^c^	1.12^c^	secondary nitrogen atoms (amides) and oxygen atoms as secondary alcohol.
P(HPMA-*co*-APMA)^d^	11,400^c^	1.10^c^	primary nitrogen atoms (amines) and secondary nitrogen atoms (amides) separated by a propyl spacer.

a Data provided by the supplier (Sigma-Aldrich) as determined by GPC.

b Protein with physical properties well documented.

c Determined by GPC in 0.05 M phosphate buffer + 0.15 M NaCl, pH 7.4 solution.

d Amino groups were determined by the TNBSA method by UV–vis spectroscopy; the content of NH_2_ is 13.4 mol %.

* *Đ*_M_ = *M*_w_/*M*_n_.

### Synthesis of Polymer and Protein-Coated AuNPs in Phosphate Buffer Solutions

AuNPs were synthesized in 10 mM phosphate buffer (PB) solutions at pH 7.4 by reduction of Au­(III) to Au(0) using either polymers or BSA protein in the dual role of reducing and stabilizing agents.^18,19^ First, 2.0 mL of 20 mM PB at pH 7.4 was added to 1.0 mL of 1.0 mg·mL^–1^ aqueous solution of BPEI, BSA, PLL, and PMeOx. In the case of PHPMA and P­(HPMA-*co*-APMA), the concentration was set higher at 10.0 mg·mL^–1^. Then, 1.0 mL of 1.0 mg·mL^–1^ chloroauric acid (25.4 mmol·L^–1^ HAuCl_4_·3H_2_O) aqueous solution was added and homogenized. Such a procedure leads to final concentrations of reactants equal to 0.25 mg·mL^–1^ for HAuCl_4_, BPEI, BSA, PLL, and PMeOx or equal to 2.5 mg·mL^–1^ for PHPMA and P­(HPMA-*co*-APMA). Depending on the polymer content, the resulting HAuCl_4_·3H_2_O:stabilizer weight ratio was 1:1 or 1:10 in 10 mM PB at pH 7.4. The chemical reactions were carried out at 75 °C until UV–vis absorption remained nearly constant over time. Solutions used in cytotoxicity and antimicrobial assays were dialyzed against PB at pH 7.4 (SpectraPor Float-a-Lyzer G2 dialysis systems; 3500–5000 g·mol^–1^ molecular weight cutoff; dialysate replaced two times over a 4 h dialysis time). The polymer- or protein-decorated AuNPs were labeled based on the stabilizer/reducing agent used in the synthesis as follows: AuNPs@BPEI, AuNPs@BSA, AuNPs@PLL, AuNPs@PMeOx, AuNPs@PHPMA, and AuNPs@P­(HPMA-*co*-APMA).

### Methods

#### UV–Vis Spectroscopy

UV–vis spectroscopy was used to calculate the molar content of TTc groups and amine groups alongside the polymer chain for the HPMA precursor and P­(HPMA-*co*-APMA). The content of TTc groups was determined in MeOH with an extension coefficient for TTc groups ε_312_ = 10,400 L·mol^–1^·cm^–1^ in triplicates. The content of hydrazide groups was determined via the 2,4,6-trinitrobenzene-1-sulfonic acid (TNBSA) assay method, as described in the literature.^20^ Kinetics of nanoparticle formation were investigated using the UV–vis profiles acquired by a Varian Cary 50 spectrometer and quartz cells with an optical path length of 1.0 cm. For catalytic activity assays, a spectrum was collected before the addition of AuNPs (i.e., before any reaction), and then the UV–vis profiles were automatically acquired by the equipment after addition of AuNP-based catalysts. In all cases, baseline corrections were made using the respective aqueous solutions as blanks.

#### Size Exclusion Chromatography

Size exclusion chromatography was used for the determination of number-average molecular weight (*M*_n_), weight-average molecular weight (*M*_w_), and dispersity (*Đ*) of polymer precursors PHPMA and P­(HPMA-*co*-APMA), as well as for determination of their purity. The HPLC Shimadzu system was equipped with a photodiode array UV–vis detector (SPDM20A, Shimadzu, Japan), refractometric detector (Optilab rEX, Wyatt, Germany), multiangle light-scattering (DAWN HELEOS II) detector, viscometry detector (ViscoStar III) (all from Wyatt Technology Co., USA), DGU-20A5R degasser, LC-20AD pump, and Superose 6 Increase 10/300 GL column (Cytiva, USA). The HPLC system was controlled by a CBM 20A unit. All samples were measured in the mobile phase (0.05 M PB + 0.15 M NaCl, pH 7.4) with a flow rate of 0.5 mL·min^–1^ for the Superose 6 Increase column.

#### Dynamic Light Scattering

Dynamic light scattering (DLS) measurements were performed using an ALV/CGS-3 platform-based goniometer system (ALV GmbH) equipped with a polarized HeNe laser (22 mW) with a wavelength of 633 nm, an ALV-7004 digital correlator, and a pair of APD-based single photon detectors. Samples were loaded into 10 mm-diameter glass cuvettes and maintained at 25 °C. The data were collected using ALV Correlator Control software with a 60 s counting time. The autocorrelation functions were acquired at 25 °C in triplicate at a 90° scattering angle (θ) and then averaged before analysis using the CONTIN algorithm incorporated into the ALV correlator control software. The resulting relaxation time distributions were further converted into hydrodynamic radius (*R*_H_) distributions by using the Stokes–Einstein equation:

$$R_{H}=\frac{k_{B}Tq^{2}}{6\pi \eta }\tau$$ 1

where *k*_B_ is the Boltzmann constant, *T* is the absolute temperature, *q* is the scattering vector, η is the solvent viscosity, and τ is the mean relaxation time. The autocorrelation functions (*g*_1_(*t*)) were also analyzed using the Cumulant fitting (second-order cumulant).^21^

$$\ln\;g_{1}\left(t\right)=\ln\;C-\Gamma t+\frac{\mu_{2}}{2}t^{2}$$ 2

where *C* is the amplitude of the autocorrelation function, Γ is the relaxation frequency (τ^–1^), and the parameter μ_2_ is known as the second-order cumulant. This approach allowed for the determination of polydispersity indexes (PDI = μ_2_/Γ ^2^).

#### Electrophoretic Light Scattering

The electrophoretic mobility of the particles investigated herein was measured using a Malvern Zetasizer Nano-ZS ZEN3600 instrument in order to evaluate their surface charge. The measurements were performed in folded capillary cuvettes (DTS1070, Malvern) containing 800 μL of the samples at 0.025 mg·mL^–1^. The ζ-potential (zeta potential) values were derived from electrophoretic mobility (*U*_E_) data using the Henry equation and assuming the Smoluchowski approximation for aqueous samples (*f*(κ*a*) = 1.5).^22,23^

$$U_{E}=\frac{2\varepsilon \zeta }{3\eta }f(\kappa a)$$ 3

where ε is the dielectric constant of the medium and η its viscosity.

#### Transmission Electron Microscopy

The polymer- or protein-coated AuNPs were imaged with a TEM microscope Talos F200X G2 (FEG-X, Brazil) using the bright-field imaging mode at accelerating voltage of 200 kV. 4 μL of each sample was deposited onto a copper TEM microscopic grid (400 mesh) coated with a thin carbon film. The grids were left to dry completely at room temperature before examination.

### Evaluation of the Catalytic Properties

The catalytic hydrogenation of *p*-nitrophenol (Nip) to *p*-aminophenol in the presence of AuNPs@macromolecule was carried in a 1.0 cm optical path-length quartz cell containing 2.0 mL of 0.1 mM Nip (2.0·10^–4^ mmol) and 1.0 mL of 0.1 M NaBH_4_ (1.0·10^–1^ mmol). Subsequently, a 50 μL aliquot of AuNPs@macromolecule solution was added to the reaction mixture in the cuvette, and the reaction was immediately monitored by following the time-dependent decrease in the UV–vis absorption at 400 nm due to the consumption of Nip reactant. The final AuNPs@macromolecule concentration in the reaction media was 0.1 ppm. Experiments were carried out at room temperature.

### MTT Cytotoxicity Assay

HeLa cancer cells were cultured in DMEM supplemented with 10% fetal bovine serum and antibiotics (penicillin 10,000 units·mL^–1^ and streptomycin 10,000 μg·mL^–1^ to prevent bacterial contamination) at 37 °C in 5% CO_2_ atmosphere. Cells at 10,000 cells/well were seeded in 96-well plates, grown for 24 h, and then incubated with AuNPs in fresh DMEM to a final volume of 100 μL. The cells were incubated with AuNPs for 24 h at 37 °C and 5% CO_2_ and then washed with fresh phosphate buffer saline (PBS). The MTT reagent solution (50 μL at 0.3 mg·mL^–1^) was added to each well and incubated for 4 h in the same atmosphere. The mitochondria of cells convert the MTT reagent to formazan crystals. The formazan crystals were then dissolved into 150 μL of DMSO, and the absorbance at λ = 570 nm was measured using a Synergy microplate reader. Positive cell viability control (C+, 100% cell viability) consisted of cells incubated with only the medium and no AuNPs in otherwise the same conditions. Negative cell viability control (C–, 0% cell viability) consisted of DMSO-filled wells. All samples were prepared in at least triplicates. Cell viability was then calculated as

$$\mathrm{CV}{\%}_{\mathrm{spl}}=100\times \frac{\left(A{®}_{\mathrm{spl}}-A{®}_{C-}\right)}{\left({\mathrm{A̅}}_{C+}-{\mathrm{A̅}}_{C-}\right)}$$ 4

where CV %_spl_ is the sample cell viability percentage, A̅_spl_ is the sample absorbance average, *A̅*_C_- is the negative control (0%) absorbance average, and *A̅*_C+_ is the positive control (100%) absorbance average.

The standard deviation of each % sample cell viability was calculated based on the propagation of uncertainty of simple functions as

$$\sigma_{\mathrm{CV}\%\mathrm{spl}}≅\mathrm{CV}{\%}_{\mathrm{spl}}\times \sqrt{{\left(\frac{\sqrt{{\sigma_{\mathrm{Aspl}}}^{2}+{\sigma_{\mathrm{AC}-}}^{2}}}{A{®}_{\mathrm{spl}}-A{®}_{C-}}\right)}^{2}+{\left(\frac{\sqrt{{\sigma_{\mathrm{AC}+}}^{2}+{\sigma_{\mathrm{AC}-}}^{2}}}{A{®}_{C+}-A{®}_{C-}}\right)}^{2}}$$ 5

where σ_CV% spl_ is the standard deviation of the sample cell viability percentage, σ_Aspl_ is the standard deviation of the sample absorbance, σ_AC–_ is the standard deviation of the negative control absorbance, and σ_AC+_ is the standard deviation of the positive control absorbance.

### Antimicrobial Activity Assay

Liquid growth inhibition assays followed the protocol described by Bulet et al.^24^ and were performed in 96-well microplates against the Gram-positive bacterium Staphylococcus aureus (ATCC 29213) and the Gram-negative bacterium Escherichia coli (ATCC 10536), both obtained from Oswaldo Cruz Foundation (Rio de Janeiro, Brazil). In peptone broth (PB; 0.5% NaCl, 1% peptone, pH 7.4), bacterial suspensions (1 × 10^3^ CFU·mL^–1^) in the mid log growth phase were treated with 2-fold serial dilutions of AuNPs for 18–20 h at 30 °C under gentle stirring. Bacterial growth inhibition was measured by absorbance at 595 nm using a Multiskan GO microplate reader (Thermo Scientific, USA). Untreated bacteria were used as the positive control for growth and as a reference for percentage growth measurements of AuNP-treated bacteria, while only the culture medium was used as the negative control for growth. The data treatment for bacterial growth is analogous to the one described for cell viability.

## Results and Discussion

### Synthesis of the AuNPs Mediated by Biomedically Relevant Macromolecules

Gold nanoparticles (AuNPs) were synthesized by using distinct polymers and one protein (BSA). The macromolecules played the roles of both reducing and stabilizing agents to yield polymer- (or protein-)­coated AuNPs in a one-pot single-step synthesis. The properties of the samples used in the present study are summarized in Table 1, with highlights to chemical structure characteristics that dictate redox potentials and steric stabilization during the synthesis of the AuNPs.

Starting with such a diverse set of macromolecules in terms of chemical structure, we first show an overview of the synthesis and properties of the resulting AuNP@macromolecules and subsequently discuss the specificities of each system. The formation of the AuNPs with the selected stabilizers was first evaluated by the characteristic red to purple color of spherical AuNP suspensions in aqueous environments. While BPEI and PLL were able to produce AuNPs in 10 mM PB at pH 7.4 and room temperature, the other polymers did not yield significant amounts of particles. For this reason and inspired by Mountrichas et al.,^9^ who demonstrated that the higher the temperature the faster the reaction, leading to more homogeneous nanoparticles, further experiments were carried out at mild temperatures. At 50 °C, AuNPs were identified after 24 h in the presence of PMeOx and 72 h in the presence of PHPMA or P­(HPMA-*co*-APMA). BSA requires at least 75 °C to produce AuNPs, and the reaction takes 48 h to occur. Therefore, all subsequent studies were conducted at 75 °C to keep such experimental variable fixed. At 75 °C, BSA protein undergoes denaturation and aggregation, with a consequent increase in the characteristic *R*_H_ from ∼4.0 to 10.0 nm.^26^ As for the other polymers, the typical *R*_H_ is below 3.0 nm (data reported elsewhere for PEI,^27^ PLL,^28^ and PMeOx^29^). After reacting with Au­(III) species to generate AuNPs@macromolecules and getting anchored to the surface as a result of the reaction process, polymer chains, henceforth comprising the outer shell of nanoparticles, may have different conformations in response to the novel surrounding environment, eventually dictating the resulting properties of the systems, as discussed hereinafter.

The reactions were clearly much faster at elevated temperatures for macromolecules that already induced the formation of AuNPs at lower temperatures, but the final properties (*R*_H_ and λ_max_) were similar, especially for PHPMA and P­(HPMA-*co*-APMA). Interestingly, PHPMA-based systems required a gold:stabilizer weight ratio of 1:10 to yield stable colloids over time; in contrast, only 1:1 was needed for the other cases.

The polymer- and protein-decorated AuNPs were then characterized by UV–vis spectroscopy, TEM, DLS, and ELS after the reactions were considered finished; such a criterion is explained below. Table 2 shows experimental data related to nanoparticle properties. In general, a typical and well-defined localized surface plasmon resonance (SPR) band was registered for all AuNPs@macromolecules, with λ_max_ ranging from 520 to 535 nm, suggesting the presence of spherical or quasi-spherical gold nanoparticles with diameters varying from 6 to 60 nm.^30^ Examination of the corresponding UV–vis spectra recorded after the reactions reached near completion (Figure 1) suggests the formation of AuNPs with rather different characteristics. In particular, AuNPs@PHPMA displayed a much lower background absorption compared to those of all other systems. AuNPs@P­(HPMA-*co*-APMA) showed the highest absorption at the SPR maximum, correlating well with the high concentration of primary nitrogen atoms (highest 1-N:Au ratio, see Table 3 below), which are known to play a predominant role in polymer-mediated AuNP nucleation and growth.

**Table 2:** Diameter Determined by TEM (*D*_TEM_), Zeta Potential (ζ), and Wavelength at Maximum Absorption (λ_max_) for AuNPs Synthesized Using Various Stabilizers According to the Labels

**polymer**	**λ**_**max**_ **(nm)**	*D*_**TEM**_ **(nm)**	ζ (mV)
BPEI	521	6.0	+13.6
BSA	525	16.8	–10.2
PLL	536	8.3	+13.3
PMeOx	526	27.5	–17.4
PHPMA	528	2.4	–7.1
P(HPMA-*co*-APMA)	535	13.3^a^	–2.0

a Feret diameter of particles with different shapes.

**Figure 1:**
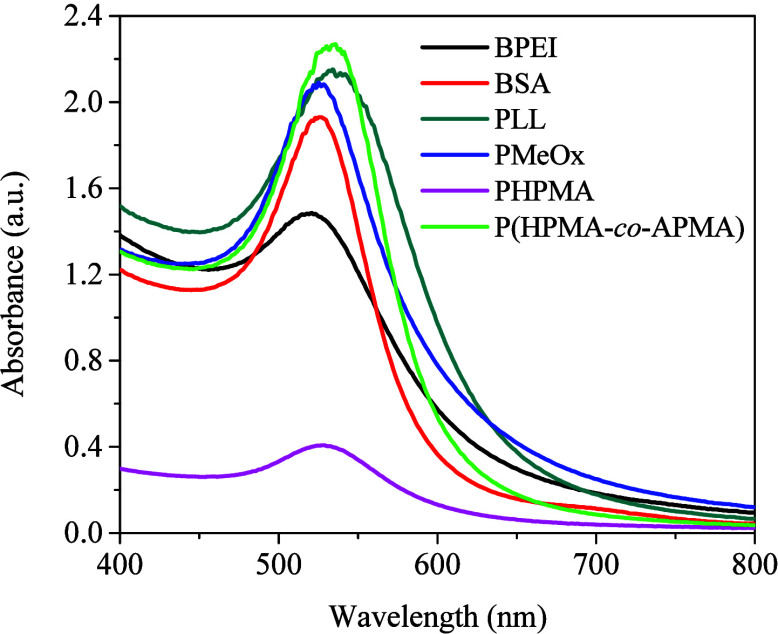
UV–vis spectra for AuNPs@macromolecules synthesized in 10 mM PB pH 7.4 solutions at 75 °C after the reactions reached near completion (near constant light absorption).

**Table 3:** Quantitative Data Obtained from UV–Vis during the Synthesis of Polymer-Coated AuNPs in 10 mM PB pH 7.4

**polymer**	**induction time**	**reaction time**	**N:Au** ^b^	1-N^c^	2-N^c^	3-N^c^
BPEI	∼3 min	40 min	9.4	2.4	4.7	2.4
BSA	26 h	52 h	3.4^d^	3.4		
PLL	50 min	3 h	6.2	3.1	3.1	
PMeOx	n.d.^e^	3.5 h	4.7			4.7
PHPMA	n.d.^e^	7 h^a^	27.3		27.3	
P(HPMA-*co*-APMA)	2 h	20 h^a^	31.4	4.1	27.2	

a Time associated to the change in the slope of absorption at λ_max_ vs time curve.

b Molar concentration ratio of nitrogen atoms (independently of the chemical group) to gold atoms.

c Breakdown of different types of nitrogen atoms (1-*N* = primary, 2-*N* = secondary, 3-*N* = tertiary) present as amine and/or amide functional groups identified in Table 1 for each polymer.

d Estimated at least one nitrogen atom per amino acid residue.

e Not detected.

Before getting insight into the course of the reactions (kinetics of AuNPs@macromolecule formation), we present the results of TEM imaging analyses of products (Figure 2). In general, number-averaged particle size analysis for AuNPs@BPEI, AuNPs@BSA, AuNPs@PLL, AuNPs@PMeOx, and AuNPs@PHPMA samples indicated that objects were spherical or quasi-spherical with diameters ranging from 6 to 30 nm, hence in good agreement with values estimated from the SPR by UV–vis. However, a predominant fraction of much smaller 2.4 nm-diameter spherical particles was found for the AuNPs@PHPMA system in addition to larger 20 nm objects inferred from the corresponding UV–vis spectrum profile (SPR band). The TEM micrograph of AuNPs@PHPMA given separately in Figure 3 for better visualization confirms the high proportion of small particles. Accurate particle counting for each population for this sample from TEM images is difficult due to the stark difference in size. Nonetheless, we concluded that the small population corresponds to about 99% of the particles within the sample. Such tiny particles, although significantly more numerous, were rightly not identified by UV–vis and DLS techniques, because, respectively, they have a characteristically low extinction coefficient and a damped SPR peak that result in a UV–vis spectrum dominated by the fraction of particles with larger size,^31^ and they scatter much less light intensity than large objects which ultimately mask the very small ones. Their existence has been further corroborated by exceptionally high catalytic and antimicrobial activities (see hereinafter).

**Figure 2:**
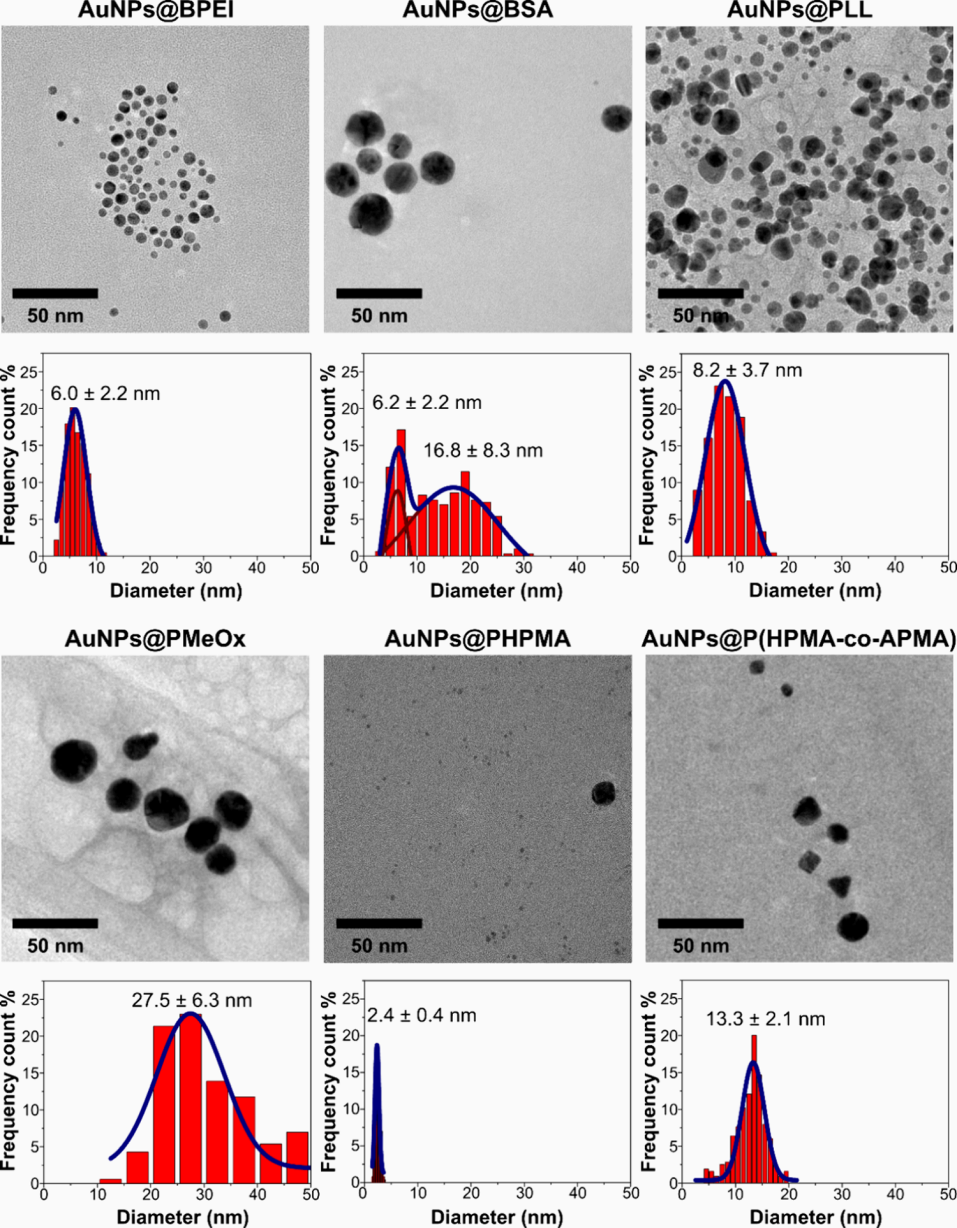
TEM micrographs and respective size distribution histograms for AuNPs@macromolecules synthesized in 10 mM PB at pH 7.4 and 75 °C, according to the labels.

**Figure 3:**
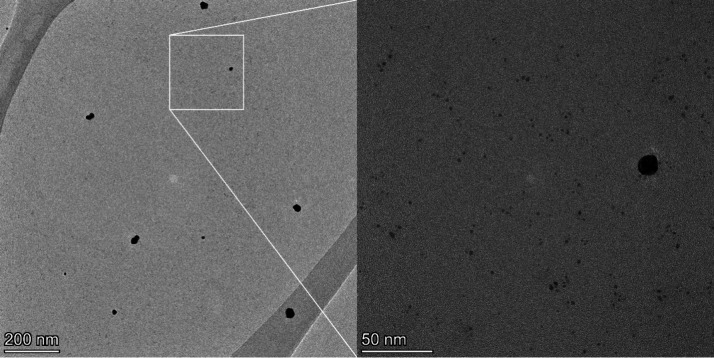
Representative TEM micrograph of AuNPs@PHPMA showing the presence of predominantly small particles (⌀ = 2.4 nm) within the sample. At lower magnification (46,000×), the small nanoparticles appear as slightly darker-than-background spots. These small particles were better visualized for size determination at a higher magnification (245,000×), when the background becomes darker due to slight beam damage to the carbon film of TEM grids. No images could be acquired at higher magnifications (<245,000×) due to considerable carbon film damage.

As for the AuNPs@P­(HPMA-*co*-APMA), different two-dimensional shapes were observed, i.e., circles with average diameter of 11.7 nm being the most frequent (54%), followed by squares of 9.5 nm average edges (29%), triangles of 11.9 nm average edges (8%), and other shapes such as 13.0 nm pentagons (3%), 15.0 nm hexagons (3%), and undefined shapes of 16.1 nm (2%) size given by the defined Feret diameter. The actual three-dimensional nature of the particles may be spheres, cubes, cuboctahedra, octahedra, tetrahedra, and truncated tetrahedra, among others.

The findings reported above prove that gold colloids with unique features are accessible using PHPMA and P­(HPMA-*co*-APMA) polymers. We further discuss this point along with the reaction kinetic data hereinafter. The surface charge of the polymer-coated AuNPs varied from −17.4 to +13.5 mV (Table 2) as determined by ELS measurements (zeta potential distribution profiles are provided in Figure S2—Supporting Information File). From the biomedical application standpoint, the zeta potential (ζ) values are in an ideal range to prevent strong nonspecific electrostatic adsorption of proteins and other species present in biological fluids.^32^ Despite the relatively low ζ values, the polymer-coated AuNPs are stable in 10 mM PB at pH 7.4, suggesting a synergic effect between steric and electrostatic stabilization. By changing the stabilizer/capping agent, particles with different surface charges could be obtained, from positive (AuNPs@BPEI, AuNPs@PLL) to negative AuNPs (AuNPs@PMeOx, AuNPs@BSA) and virtually neutral AuNPs@PHPMA and AuNPs@P­(HPMA-*co*-APMA). The cationic nature of AuNPs@BPEI and AuNPs@PLL are expected from the characteristic aqueous solution behavior of amino groups around the metallic cores, as well as predictable anionic nature of AuNPs@BSA, since above the isoelectric point BSA carries negative net charge.^25^ The case of PMeOx is different. As a nonionic polymer, the most negative net charge of AuNPs@PMeOx among all the samples is attributed to a combination of chemical transformations (oxidation) of polymer chains adjacent to the metal surface and organization of potential determining and/or indifferent ions within the electrical double layer around the particle. The same applies for AuNPs@PHPMA, although the resulting particles exhibit a less negative net surface charge. Even less anionic are the AuNPs@P­(HPMA-*co*-APMA) particles probably due to the protonation of a fraction of primary amine groups of the APMA repeating units (p*K*_a_ of APMA ∼ 9.1 ^33^).

The nanoparticle formation was investigated by UV–vis spectroscopy, monitoring the evolution of spectra during the synthesis. The wavelength at maximum SPR absorbance is mostly unchanged during this process, thus allowing kinetic data to be obtained from corresponding changes of absorption at λ_max_ as a function of the reaction time (Figure 4). Raw spectral data and kinetic profiles for each sample are provided in the Supporting Information File (Figures S3–S8).

**Figure 4:**
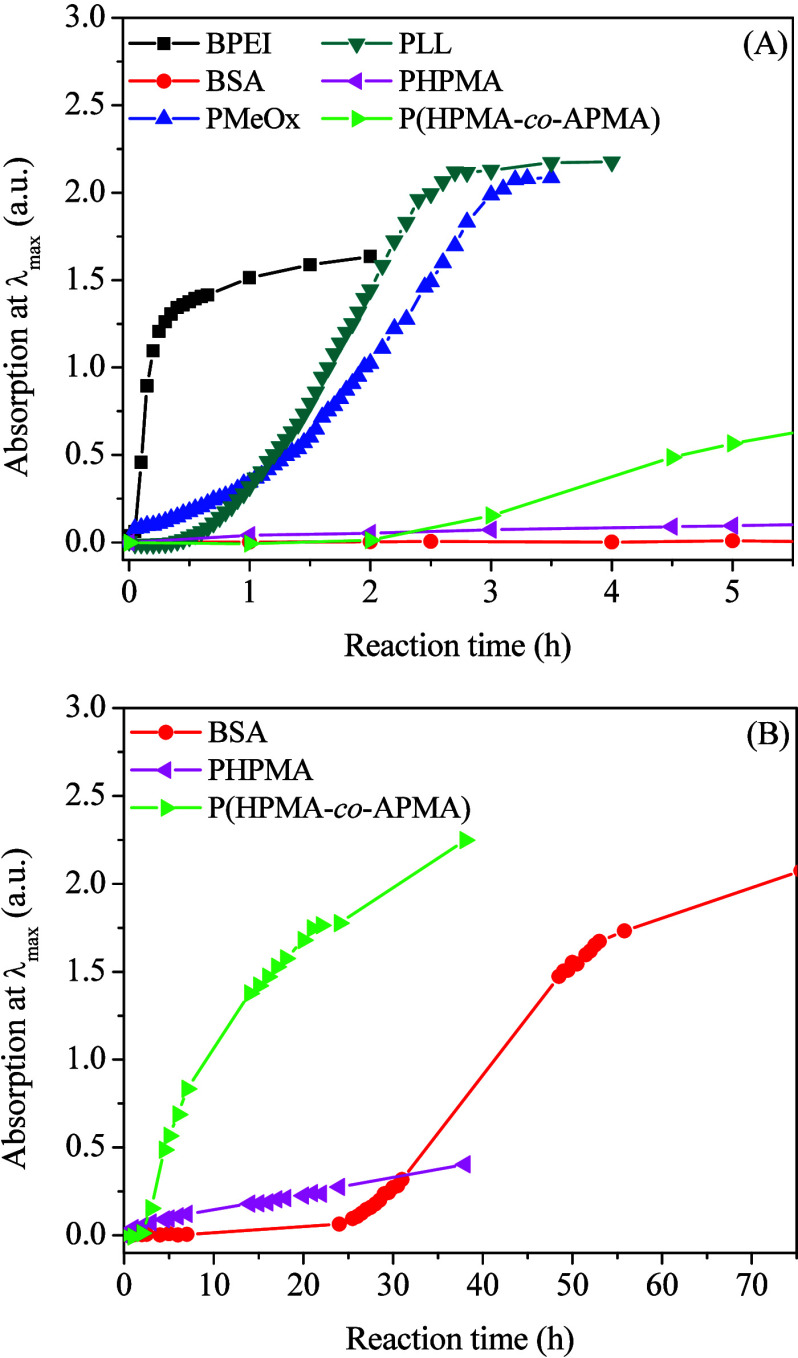
Kinetics profiles for the synthesis of AuNPs@macromolecuels in 10 mM PB at pH 7.4 at 75 °C (A). Data for long reaction times in selected cases (B).

The four-step process of AuNP formation formerly proposed by Polte et al.^34^ for citrate chemistry is widely accepted. As reviewed by Thanh et al.,^35^ nucleation occurs first yielding very small particles, which grow in the second step through either Ostwald ripening or coalescence. Then, self-sharpening growth by diffusion takes place before a final step, where the particle size increases rapidly due to the rapid consumption of the gold ions left in the solution. The latter is an autocatalytic reaction on the surface of the nanoparticles, which manifests as a curve of a sigmoidal shape. This curve can be visually divided into three parts: (*i*) induction, while gold ions are reduced to gold atoms until a critical atom concentration is reached, and then the first gold nuclei start to be formed (nucleation: step 1); (*ii*) particle growth (steps 2 to 4), where the formed gold clusters grow through different mechanisms, as previously mentioned; and (*iii*) saturation, when the reaction is reaching its equilibrium and the final features of AuNPs are achieved; thus, the UV–vis spectrum remains nearly constant over time—the reaction was considered completed when such a condition was fulfilled. The induction time and total reaction time are summarized in Table 3 along with the stoichiometry ratio between nitrogen and gold atoms.

The kinetic curve recorded for AuNPs@BPEI confirms the strong effect of the reaction temperature. The induction time decreased from 30 min at room temperature^7^ to just 3 min at 75 °C, and the reaction reached completion after 40 min instead of 10 h. Interestingly, particles obtained in this study were more uniform, although ostensibly larger (6 nm as compared to 3.6 nm) than previous attempts at room temperature as deduced from histograms. In comparison, the formation of AuNPs@PLL, which also features primary and secondary amines, was slower, requiring approximately 50 min of induction time before particle growth, at a slow rate as inferred from the slope of the curve. Even though the autocatalytic processes are different since particles are distinct, the [N]:[Au] stoichiometric ratio is lower in the case of AuNPs@PLL (see Table 3). Thus, we speculate that the lower reactant concentration is also a factor contributing to a slower reaction for AuNPs@PLL compared to that for AuNPs@BPEI.

The profile for AuNPs@PMeOx did not display a clear induction period. Instead, the absorbance gradually increased in the early stages of the reaction before getting slightly faster after ∼1.0 h and then continuing at a near constant rate from 1.5 up to 2.5 h (see the almost constant slope). We attribute this behavior to the delocalization of the lone pair of electrons on the nitrogen atoms that renders PMeOx a poor nucleophile, hence with a lower ability to interact with gold and/or donate electrons. Nonetheless, AuNPs@PMeOx slowly grew, yielding the largest particles among all the systems. The number of nuclei in the first step of the process should be much lower in the case of AuNPs@PMeOx than AuNPs@PLL, because after the nucleation step, the growth mediated by PLL was faster (steeper curve) but generated smaller particles (more particles of smaller size).

The formation of AuNPs@BSA was rather intriguing. Very long induction (26 h) and reaction times (52 h) were observed. Despite having the characteristic sigmoidal curve of nanoparticle formation consistent with an autocatalytic process, this being the reason for the sudden acceleration after a long nucleation period, this system also presented a nearly linear absorbance increase in the closing stages of the reaction. Two particle size populations were evidenced by TEM (Figure 2), which had diameters of 6.2 and 16.8 nm, respectively, corresponding to 16 and 84% of the objects.

The synthesis of AuNPs@PHPMA and AuNPs@P­(HPMA-*co*-APMA) was carried out at a higher polymer concentration (2.5 mg·mL^–1^ instead of 0.25 mg·mL^–1^) to attain stable colloids. Upon increasing the polymer concentration, the [N]:[Au] stoichiometric ratio was obviously much higher (3-fold or more) for these two cases, as given in Table 3. The kinetic plot for AuNPs@PHPMA appearing in Figure 4 (main panel and inset) is based on absorption values at λ_max_ that originate from a low extinction coefficient and a damped plasmon resonance peak of small particles, as mentioned above.^31^ For a better visualization, refer to Figure S7 (Supporting Information). In an environment with high concentration of secondary amide groups, the onset of AuNPs@PHPMA formation is almost immediate, exhibiting a linear increase afterward, without reaching a detectable plateau in this case. Post-reaction analysis confirmed that 99% of the objects within the sample are small particles (⌀ = 2.4 nm). Similarly, small particles were synthesized in the presence of thiol-terminated poly­(ethylene glycol) using a strong reducing agent.^36^ It is very important to note that the characteristic sulfur-containing end groups of RAFT-made PHPMA and P­(HPMA-*co*-APMA) polymers were replaced by isobutyronitrile motifs (Figure S1). Therefore, the passivating effect against nanoparticle growth imparted by polymeric thiols is ruled out here. Nevertheless, nitrile groups do interact with trivalent^37,38^ and zerovalent^39^ gold species by σ- or π-coordination, with AuNPs being reported as convenient catalysts for the reduction of nitriles to amides.^39,40^ From a coordination chemistry perspective, the interaction nitrile–gold is stronger than the amide–gold analog. In the present case, the stoichiometry of [CN]:[Au] ∼ 0.7 is thus not negligible. We checked whether this interaction resulted in changes in the UV bands assigned to ligand-to-metal charge transfer (below 350 nm) but found no evidence in the 200–350 nm-wavelength range.

At this point, combining all the results of the present study, the macromolecular characteristics of the sample, and previous knowledge of the mechanisms of nucleation and growth of gold nanoparticles,^34–36^ we postulate the following hypothesis: the presence of coordinating nitrile ([CN]:[Au] ∼ 0.7) and amide ([2-N]:[Au] ∼ 30) functional groups at high stoichiometric proportion enhances particle nucleation (first step in Polte’s mechanism), so that the same amount of gold is distributed among a larger number of small particles;^36^ then, effective steric stabilization blocks coalescence^41^ attributed to both high polymer concentration and strong interactions between gold clusters/nuclei and nitrile motifs at the α- and ω-end groups of PHPMA, similarly to thiol-terminated polymers; the binding of terminal nitriles to AuNPs may include upright σ interaction and flat-lying π interaction, as inferred from the interaction of terminal alkynes with gold^42^ and other metals.^43^ The introduction of primary amine groups of higher nucleophilicity, known to function better as reducing and capping agents, unlocks this situation. The kinetic profile of AuNPs@P­(HPMA-*co*-APMA) recovers the typical sigmoidal behavior, although particles of distinct shapes were detected. We have indeed investigated this using a wide library of P­(HPMA-*co*-APMA) copolymers to properly clarify such an initial hypothesis.

### Evaluation of the Catalytic Activity

The catalytic hydrogenation of *p*-nitrophenol (Nip) to *p*-aminophenol (Amf) in the presence of AuNPs@macromolecules was used as a tool to get insight into physicochemical aspects related to the conformation and the interaction of polymer chains providing steric stability to gold colloids, as well as the dynamics of small-molecule diffusion through the stabilizing layer, and their adsorption and desorption onto the metal surface. As Nip substrate can induce surface restructuring,^44^ these catalytic studies also highlight the influence of the chemical nature of the AuNP stabilizer on the gold surface chemistry.

Under the experimental conditions used in this study, the aqueous dissociation equilibrium is shifted toward the *p*-nitrophenolate ions, which yield the solutions an intense yellow color with absorbance centered at 400 nm. The reduction of *p*-nitrophenolate to *p*-aminophenolate is thermodynamically favorable but kinetically hindered, remaining stable over hours. The addition of a catalyst then allows for the fast reduction to take place, and the reaction can be followed by the decrease in the absorption band at 400 nm due to the consumption of the Nip reactant. In the presence of a large molar excess of NaBH_4_, the six-electron transfer process is best described by a pseudo-first-order law.^44,45^ Therefore, the apparent rate constant (*k*_app_) and the induction time (*t*_0_, if present) can be derived from plots of the natural log of the corrected absorbance at 400 nm—ln (*A*_t_/*A*_0_)—versus elapsed reaction time. These plots are depicted in Figure 5, and the respective *t*_0_ and *k*_app_ values are shown in Table 4, which also include remarks to improve the reader experience. As-obtained *k*_app_ values normalized to the average surface area of the AuNPs are denoted as *k*_aap_ values. Important for these experiments is the accurate AuNP concentration, which was first determined by ICP-MS and compared to the concentration estimated by the UV–vis spectroscopy, as previously reported.^19^ The concentration estimates by UV–vis and ICP-MS were in good agreement, allowing for a double check before each experiment.

**Figure 5:**
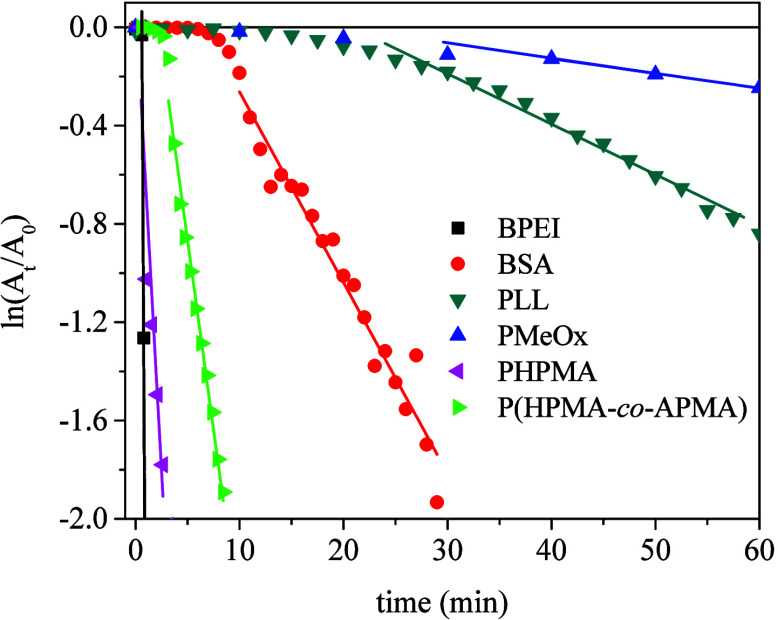
Pseudo-first-order plots for AuNPs@macromolecule-catalyzed hydrogenation of Nip as monitored by UV–vis absorbance at 400 nm ([Nip] = 6.6 × 10^–5^ M, [NaBH_4_] = 3.3 × 10^–2^ M, *C*_Au_ = 0.1 ppm, 25 °C).

**Table 4:** Selected Properties of AuNP@macromolecule Catalysts and Apparent Rate Constant (*k*_app_) for the Hydrogenation of Nip

**capping agent**	*t*_**0**_ **(** *s* **)**	*k*_**app**_ ×10 ^**2**^ **(**s^‑1^ **)**	*k*_**aap**_ ×10^**3**^ **(**s^‑1^·m^‑2^ **)**	**summary of catalyst characteristics**
BPEI	35	5.3	3.35	⌀ = 6.0 nm; ζ = +13.5 mV; well-dispersed particles
PLL	1254	0.034	0.029	⌀ = 8.3 nm; ζ = +13.3 mV; imaging and scattering techniques show particle agglomeration
BSA	396	0.13	0.230	⌀ = 6.2 nm; ζ = −10.2 mV; well-dispersed particles
PMeOx	1128	0.01	0.029	⌀ = 27.5 nm; ζ = −17.4 mV; well-dispersed particles
PHPMA	n.d.^a^	1.30	0.33	⌀ = 2.4 nm; ζ = −7.1 mV; very small well-dispersed particles
P(HPMA-*co*-APMA)	132	0.52	0.73	⌀ = 13.3 nm; ζ = −2.0 mV; only half of the particles are quasi-spherical, as imaging revealed other shapes

a Not detected.

It clearly stands out from Figure 5 and Table 4 that the AuNPs@macromolecule systems herein investigated exhibit quite different catalytic activity among themselves, reflecting the effects of functional groups, polymer chain conformation, and gold–polymer coordination.

The reactions proceeded much faster in the presence of AuNPs@BPEI, AuNPs@PHPMA, and AuNPs@P­(HPMA-*co*-APMA) catalysts than in other cases, as confirmed from the slope of kinetics graphs and the corresponding *k*_app_ values, which are comparable to previous reports.^18,46^ Also, these systems exhibited either a very short induction time or no induction time *t*_0_. Such an initial period of time has been found for several metallic nanoparticle catalysts applied for hydrogenation of Nip to Amp, while its origin remains debatable and possibly involves surface restructuring such as leaching of gold atoms induced by Nip binding, reaction with borohydride such as the transfer of a surface-hydrogen species to metal nanoparticles, or diffusion-controlled process of substrate adsorption onto the nanoparticle surface.^44,47,48^ The interplay between induction time and kinetics reveals a structure–activity relationship with the soft shell (see below).

AuNPs@BPEI clearly is the most effective catalyst. The remarkable activity can be explained by the easier diffusion of the anionic reactants (*p*-nitrophenolate and BH_4_^–^ ions) and products (*p*-aminophenolate ions) into and out of the positively charged polymer coating brush. In addition, BPEI chains are tethered to the metallic core surface, representing the so-called spherical cationic polyelectrolyte brushes (i.e., a metallic core and a cationic corona brush), which are more expanded with respect to the equivalent neutral counterparts (or highly screened equivalent polyelectrolyte chains). In such a conformation, the active gold surface is more easily accessible, yielding faster reactions.

The equally excellent results obtained using PHPMA-coated AuNPs are attributed to the high surface area that is inherent in very small particles. For the sake of comparison, under the experimental conditions of this study, the active surface area is ∼40 m^2^ for the PHPMA system as compared to only ∼16 m^2^ for the BPEI, i.e., nearly half of it. The small particle size also implies that a small number of polymer chains are anchored to the surface, and therefore, the stabilizing shell is thin due to geometric constraints. In turn, this facilitates access to the active core, with a phenomenon being enhanced by the large catalyst area, almost twice. Another contribution should also be taken into account: the interaction between the PHPMA capping agent and metallic gold is weaker than the interaction between BPEI and metallic gold, because the resonance in amide groups delocalizes the free pair of electrons on the nitrogen atoms that normally interact with gold in the case of amines. The interaction of PHPMA with the metallic core through a small amount of nitrile end groups (discussion above) as compared to concentrations of amide groups and Nip substrate does not affect the catalytic process. As a consequence, the substrate can easily replace the coordinating sites occupied by weakly bound PHPMA, yielding to faster processes.^49^ Combining all of the aspects mentioned so far, the net result is that the substrate rapidly reaches and attacks the catalyst surface, thus eliminating the induction period. The net negative charge of AuNPs@PHPMA (ζ = −7.1 mV) seems not to be a hindrance.

Upon changing the composition of the PHPMA homopolymer to the P­(HPMA-*co*-APMA) copolymer featuring primary amine groups, the behavior is quite the opposite. Being so, it further corroborates the reasoning supporting PHPMA results just mentioned above. First, an induction period appears due to a strong competition for coordinating sites of the gold surface between amino-functionalized polymer and the substrate. It is also interesting to note that the total surface area for P­(HPMA-*co*-APMA) was only ∼7 m^2^, as particles were not only bigger and with a thicker stabilizing layer but also only half of them are quasi-spherical, as revealed by TEM imaging. For this reason, we think that these data can be considered sufficient for explaining the catalytic behavior but no more.

AuNPs@PLL and AuNPs@PMeOx were the least catalytically active among all of the samples. At first, we expected AuNPs@PLL to perform similarly to AuNPs@BPEI as it consists of particles with comparable size, net cationic charge, and chemical groups. However, we consistently identified a tendency to form large aggregates while performing DLS experiments in solution and TEM imaging upon drying (see Figure S9 in the Supporting Information File). Good agreement between the TEM diameter for these agglomerates (∼200 nm) and the DLS diameter (∼235 nm) shows that these structures are indeed present in AuNPs@PLL samples in solution. No such aggregation was observed for the other samples during the TEM imaging.

The least effective was the AuNPs@PMeOx catalyst. The poor performance cannot be explained solely by the fact that big particles imply a small active surface area. The relatively high net anionic charge of AuNPs@PMeOx nanostructures (ζ = −17.4 mV) may hinder the diffusion of anionic species participating in the reduction reaction.

### Evaluation of Cytotoxicity and Antimicrobial Activity

The AuNP-induced cytotoxicity is an intrinsic complex problem. Not only is the understanding of its molecular origin challenging^50^ but also several physical chemical parameters can suddenly become relevant, especially when comparing systems whose properties cannot be independently controlled such as particle size and chemical composition of the polymeric coating in the present case. There are multiple mechanisms that lead to metallic nanoparticle toxicity. Current knowledge indicates that cell death is most often associated with reactive oxygen species (ROS) within the cell environment;^51^ meanwhile, other pathways remain open. Particles reach that space, undergoing endocytosis in a process governed mainly by nanoparticle size and surface chemistry. The number of particles having such a fate is obviously of due relevance, as intracellular accumulation can trigger cellular and acellular responses, culminating in cell death. Therefore, it is mandatory to look at these three aspects (size, number, and surface chemistry). It is indeed a puzzle that urges for a more intelligible scenario, as stated by Fratoddi et al.,^52^ and we carefully considered that advice.

Keeping this in mind, we addressed the cytotoxicity of as-synthesized AuNPs@macromolecules, limiting the scope of the study to the viability of HeLa cells. Figure 6 shows the viability of HeLa cells incubated in the presence of different AuNPs@macromolecules as evaluated by the MTT assay. While we choose to carry out dose-dependent experiments in terms of mass concentrations (ppm), the results are also interpreted based on numerical particle concentrations (number of particles per volume unit).

**Figure 6:**
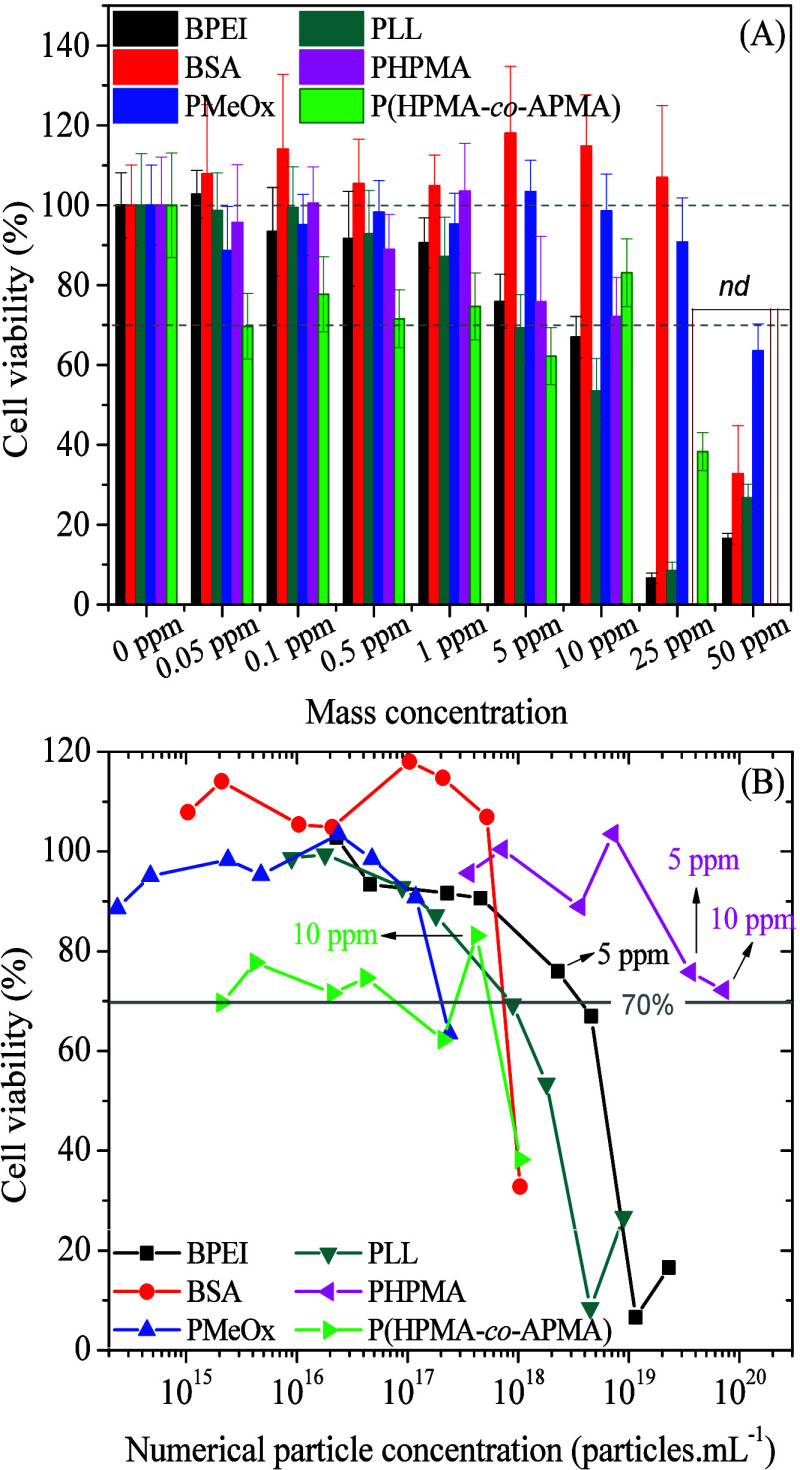
Dose-dependent viability of HeLa cells incubated with AuNPs@macromolecules of interest to this study, as indicated, represented in terms of mass concentration (ppm) and numerical particle concentrations (B). nd—not determined.

Considering that reductions in cell viability up to 30% can be regarded as noncytotoxic environment, all samples were found noncytotoxic up to 5.0 ppm. Under these conditions, the numerical particle concentration of AuNPs@PHPMA (∼3.6 × 10^19^ particles·mL^–1^) is 15-fold higher than for instance AuNPs@BPEI (∼2.3 × 10^18^ particles·mL^–1^), highlighting the exceptionally good biocompatibility of the former. In fact, cell viability of AuNPs@PHPMA is still acceptable (∼72%) even at 10.0 ppm (7.2 × 10^19^ particles·mL^–1^, also equivalent to 120 nM). At this concentration, the number of particles is 30-fold higher than at the highest noncytotoxic concentration of AuNPs@BPEI. Cells remain viable at 10.0 ppm for AuNPs@P­(HPMA-*co*-APMA) too, but it should be noted that the number of particles (4.2 × 10^17^ particles·mL^–1^) is only ca. 1% compared to AuNPs@PHPMA and ∼18% of AuNP@BPEI, both at 5.0 ppm. Upon increasing the concentration to 25 ppm, AuNPs@P­(HPMA-*co*-APMA) becomes cytotoxic in spite of the fact of much fewer particles as a nearly equal numerical particle concentration is found in systems containing 25 ppm AuNPs@P­(HPMA-*co*-APMA) and 0.15 ppm AuNPs@PHPMA. We hypothesize that this phenomenon could be ascribed to the remaining content of amino groups in the copolymer, which can have the cytotoxic effect by the interaction with the membranes of HeLa cells.

This finding puts forward the possibility that functionalizing a polymer of well-known biocompatibility such as PHPMA with a comonomer featuring primary amino groups can lead to striking drawbacks in this regard. Indeed, the almost neutral AuNPs@P­(HPMA-*co*-APMA) particles (ζ = −2.0 mV) turn cytotoxic at near the same quantitative number as AuNPs@BPEI and AuNPs@PLL, which are positively charged particles (ζ ∼+13 mV).

The investigation of the molecular origin of these effects is beyond the scope of this study. Nonetheless, the cytotoxicity of cationic species is commonly attributed to electrostatic interactions with the negatively charged biomolecules present in cell membranes.^53^ This has been observed when evaluating the cell cytotoxicity of the polymers themselves (Figure S10 and Supporting Information File) where PLL, BPEI, and P­(HPMA-*co*-APMA) are clearly more toxic than the others. The systems share the common characteristic of having primary amino groups at the outermost interface with the surroundings and also a small hydrophobic moiety neighboring the positive charge, which can induce lytic interaction with plasma membranes, as reported by Lee et al.^50^ Apart maybe from the abundance of exposed amino groups, the aqueous dissociation constants of PAPMA,^33^ BPEI,^54^ and PLL^55^ are similar. Last but not least, the cytotoxicities of AuNPs@BSA and AuNPs@PMeOx do not differ significantly among other samples when the numerical particle concentration is factored in.

The antimicrobial activity of the AuNPs@macromolecules was evaluated by liquid growth inhibition assay using E. coli and S. aureus as Gram-negative and Gram-positive bacteria, respectively (Figure 7). AuNPs@BPEI presented a total growth inhibition against both strains in the concentration range tested, while AuNPs@PHPMA presented total antibacterial activity against E. coli and dose-dependent antibacterial activity against S. aureus. The stronger cell wall of Gram-positive bacteria as compared to the thin cell wall of Gram-negative bacteria imparts higher resistance to nanoparticle mechanisms of action, thus requiring higher concentrations to achieve the same effect.^56^ The same rationale presented above in terms of numerical nanoparticle concentrations also applies to antioxidant activity. Small particles tend to be more toxic than large counterparts due to an augmented production of ROS that ultimately damage or inactivate bacterial cellular functions.^56^ On the other hand, the remaining AuNPs (AuNPs@PLL, AuNPs@PMeOx, AuNPs@BSA, AuNPs@P­(HPMA-*co*-APMA) were not effective antimicrobial agents in the concentration range evaluated, most times leading to bacterial growth above 100%, that is, above the control. To test if the macromolecules present in the AuNPs that induce bacterial growth could potentially be used as an extra source of nutrients by bacteria in the medium, we performed the inhibition assay in contact with only the macromolecules at similar concentrations to those contained in the AuNP samples. The results shown in Figure S11 (Supporting Information File) highlight that, in most cases, the macromolecules did not induce higher than control growth (at least not to the same degree). This discrepancy could be due to a more locally concentrated macromolecule amount over bacteria when AuNPs interact with them. Nevertheless, these results highlight that these AuNPs are not effective antimicrobial agents. Additionally, the evaluation of bacterial growth inhibition by the macromolecules also demonstrated that the antibacterial activity of AuNPs@BPEI and AuNPs@PHPMA is not associated with the polymers alone, which did not lead to bacterial growth inhibition by themselves, suggesting that antibacterial activity of these two AuNPs can be due to a synergistic effect between the metallic nanoparticles and their polymer coating.

**Figure 7:**
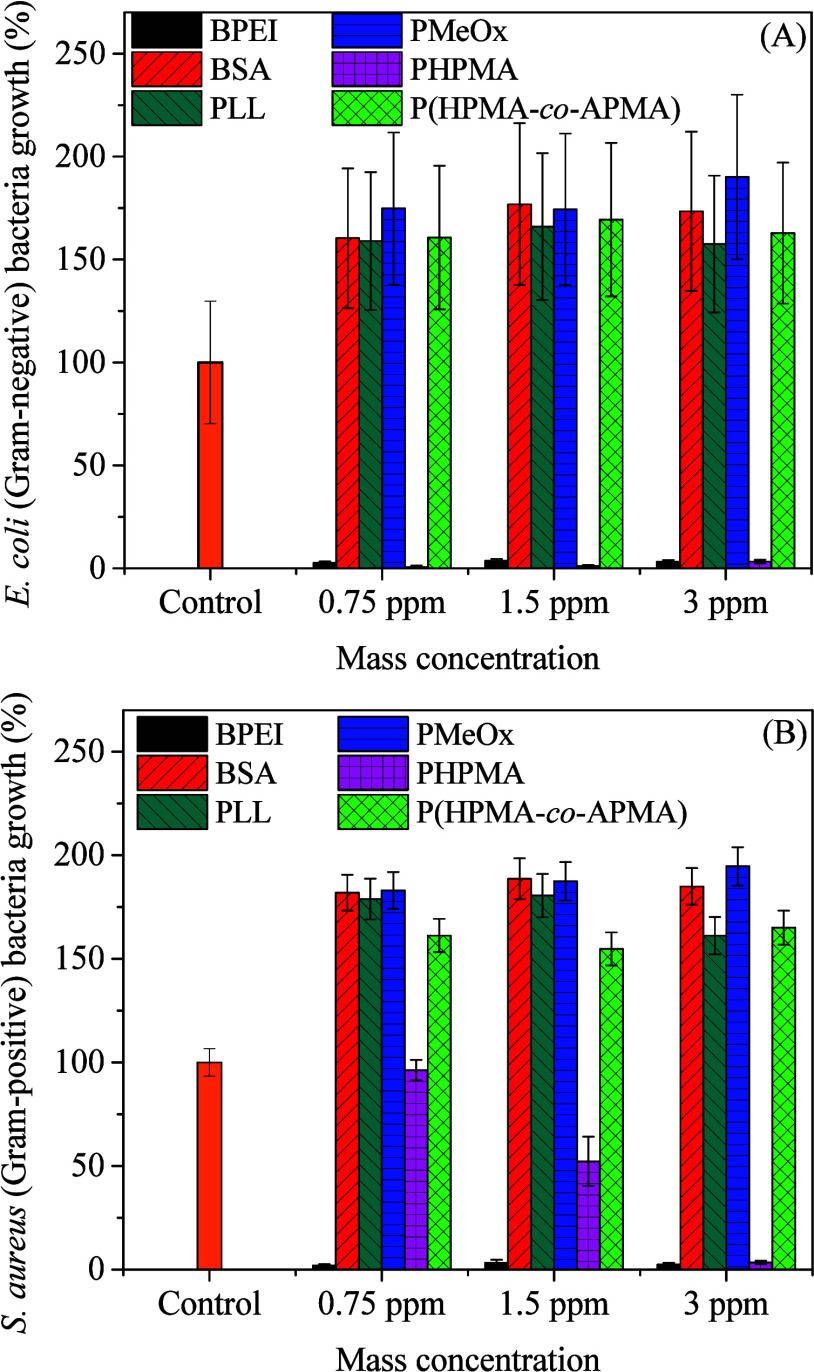
Bacterial growth of Gram-negative E. coli (A) and Gram-positive S. aureus (B) when in contact with distinct AuNPs at different concentrations according to the labels.

## Conclusions

Following our early investigations regarding the direct synthesis of coated gold nanoparticles mediated by polymers with amine groups,^6,7,18^ we herein discovered that the same approach can be expanded to manufacture AuNPs using a variety of nitrogen-containing polymers. Macromolecules featuring all types of primary, secondary, and tertiary amino groups, as well as primary and secondary amide groups, can successfully mediate AuNP formation by functioning simultaneously as a reducing and capping agent in one-pot two-reactant chemical processes in aqueous environments at mild temperatures. The reaction temperature is indeed a critical parameter. While polymers carrying primary amino groups are generally able to produce AuNPs already at room temperature, amide-containing analogs require some heating. We found that 75 °C is an adequate reaction temperature for all of the macromolecules selected for this study. One of the most relevant points of this discovery is the fact that this has been achieved by using polyamides of great interest in biomedical applications (PMeOx and PHPMA, for instance). The kinetics of nanoparticle formation revealed disparate behaviors, yet all polymers led to the formation of nearly spherical particles. The chemical features of the polymer chains induce the formation of AuNPs with distinct sizes; quite large particles were produced in the presence of PMeOx, and notably small assemblies were generated using PHPMA, which truly imparted outstanding catalytic and antimicrobial activity to such a system while keeping low cytotoxicity. Currently, we do not have experimental evidence clarifying the (macro)­molecular origin for the formation of such small particles, but a hypothesis that takes into consideration terminal groups is postulated from the perspective of the current knowledge on polymer-mediated AuNP synthesis. As a vision of future work, we are already conducting further experiments using a wider library of PHPMA and P­(HPMA-*co*-APMA) copolymers (with various molecular weights, APMA contents, and unreactive terminal groups) to properly explain the matter. The discoveries of this study might lead to significant advances toward the facile and ready-to-use production of AuNPs coated with polymers of biomedical interest that find a myriad of applications.

## Supplementary Material



## Notes

Scheme for the synthesis of the PHPMA homopolymer and P­(HPMA-*co*-APMA) copolymer, GPC chromatograms showing ω-end group replacement, raw spectral data, kinetic profiles, TEM, ELS, and DLS data for the synthesized AuNPs@macromolecules (PDF)
