# Supplementary material for: Mild-Temperature Synthesis of Gold Colloids with Unique Features Mediated by Polymers of Biomedical Interest
Source: ACS Omega. 2025 May 22;10(21):21701–14. doi: 10.1021/acsomega.5c01123 (PMC12138685; doi:10.1021/acsomega.5c01123)
Supplement: Supplementary file 1 [file ao5c01123_si_001.pdf]

Supporting Information for

Mild Temperature Synthesis of Gold Colloids with Unique Features Mediated by Polymers of Biomedical Interest

Gabriela Borba Mondo,[a] *Caroline Arana da Silva Ribeiro,*[a] *Alžběta Turnovská,*[b] *Michaela Hrochová,*[b] *Tomáš Etrych,*[b] *Fernanda Dias da Silva,*[a] *Cristiano Giacomelli,*[c] *and Fernando C. Giacomelli* [a,*]

[a] Centro de Ciências Naturais e Humanas, Universidade Federal do ABC, Santo André, 09280-560, Brazil

[b] Institute of Macromolecular Chemistry, Czech Academy of Sciences, Prague, 162 00, Czech Republic

[c] Departamento de Química, Centro de Ciências Naturais e Exatas, Universidade Federal de Santa Maria, Santa Maria, 97105-900, Brazil.

* Corresponding author: fernando.giacomelli@ufabc.edu.br

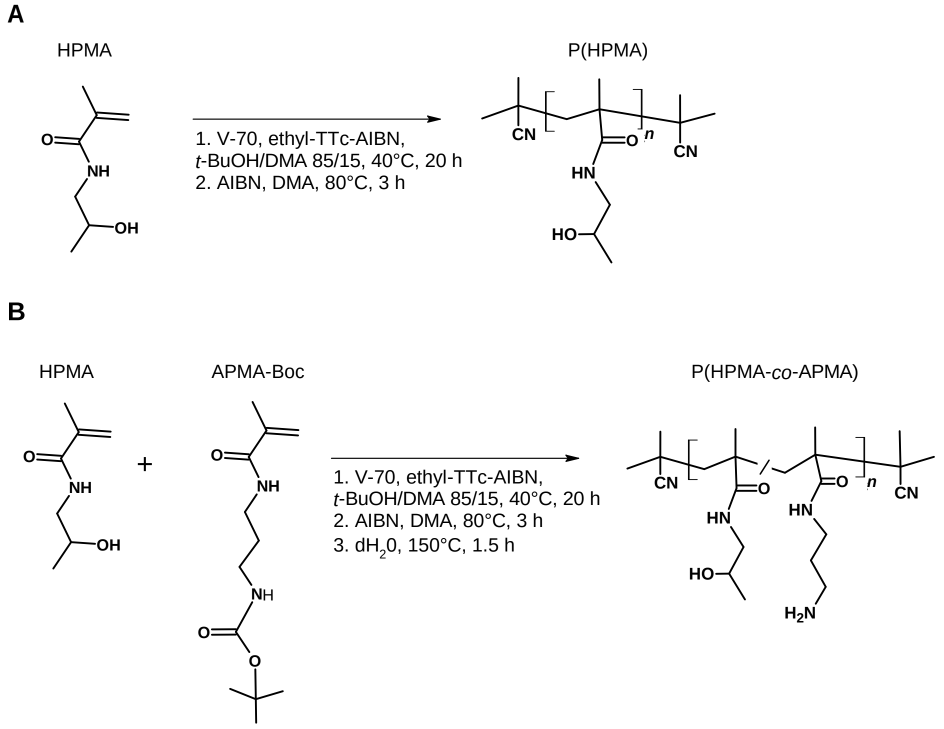

Scheme S1: Synthesis of PHPMA homopolymer (A) P(HPMA-*co*-APMA) copolymer (B).

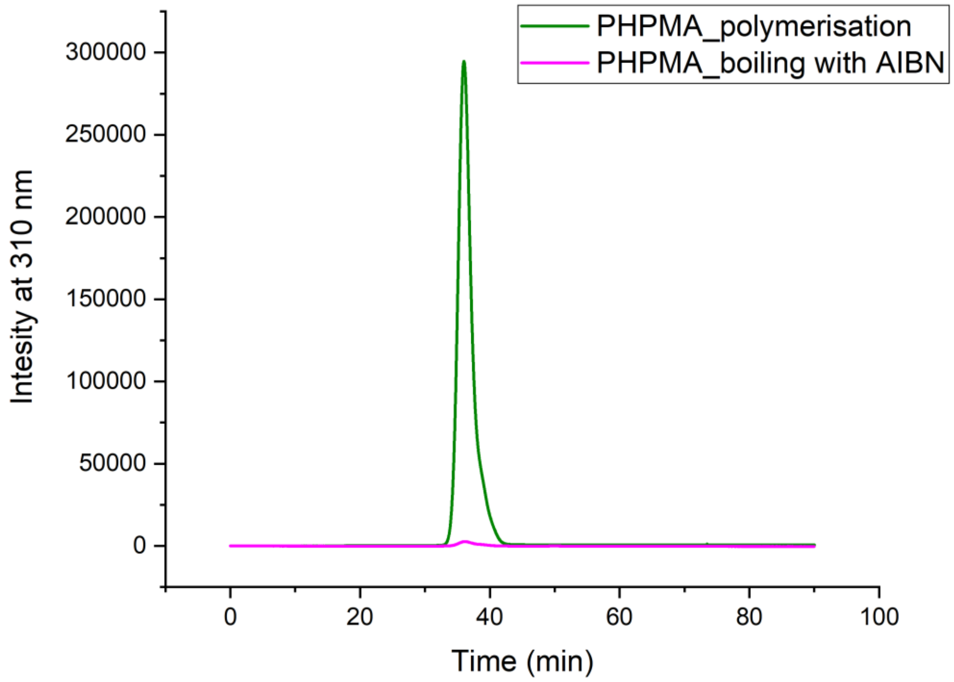

Figure S1. GPC chromatograms recorded using UV detector at 310 nm showing successful (98%+) trithiocarbonate ω-end group replacement by an isobutyronitrile motif.

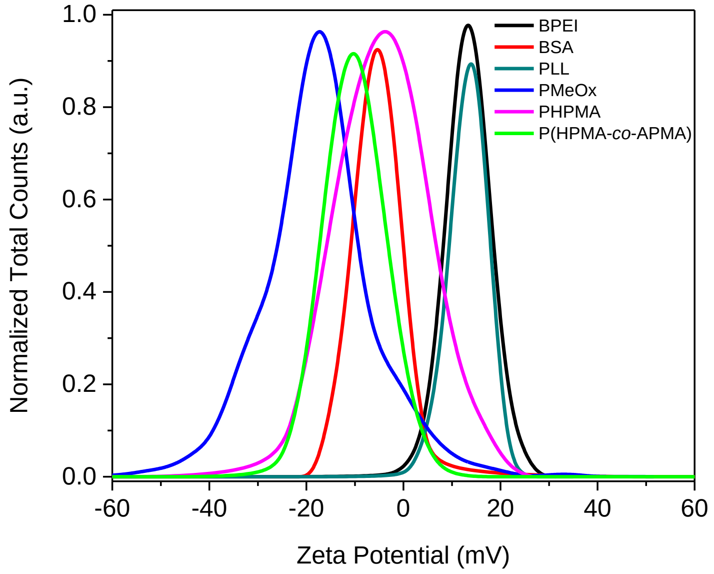

Figure S2. Zeta potential distributions revealed by electrophoretic light scattering measurements for all systems investigated in this study.

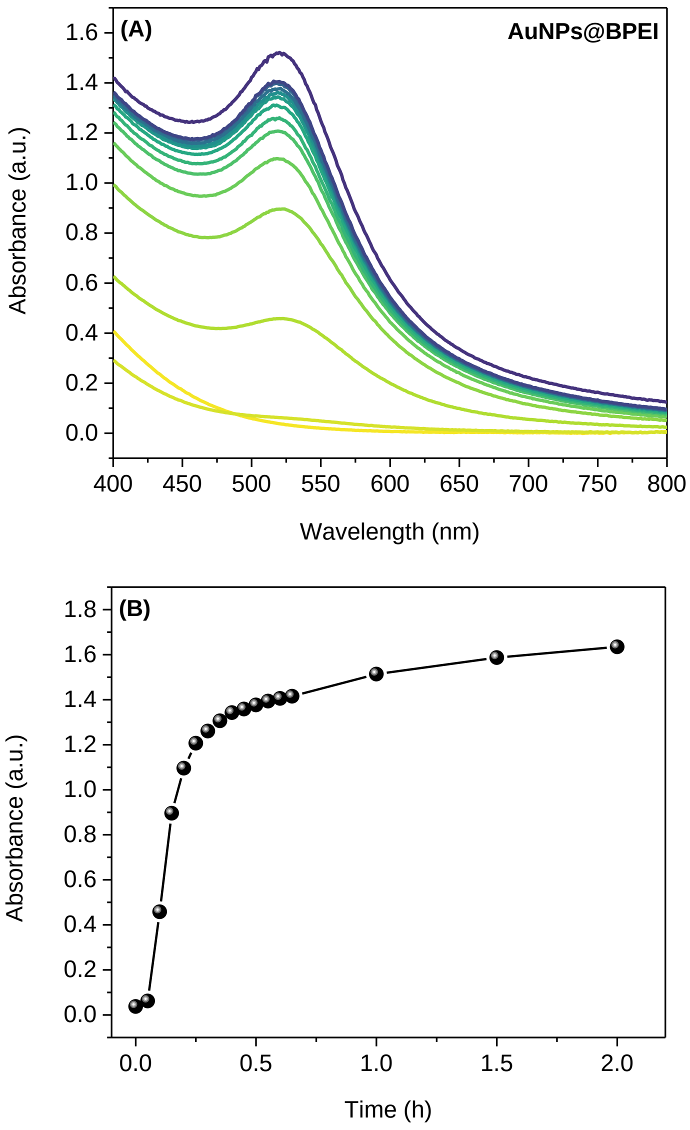

Figure S3: Evolution of UV-vis spectra for AuNPs@BPEI in 10 mM PB at pH 7.4 at 75°C (A) and respective values of absorbance at λ_{max} nm as a function of time (B).

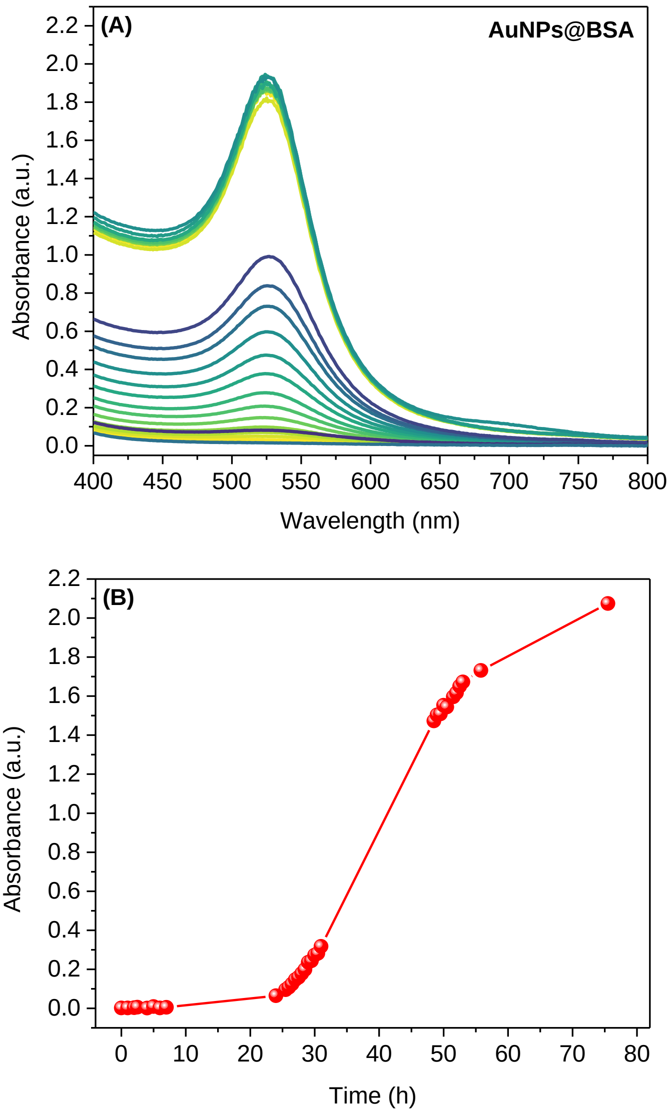

Figure S4: Evolution of UV-vis spectra for AuNPs@BSA in 10 mM PB at pH 7.4 at 75°C (A) and respective values of absorbance at λ_{max} nm as a function of time (B).

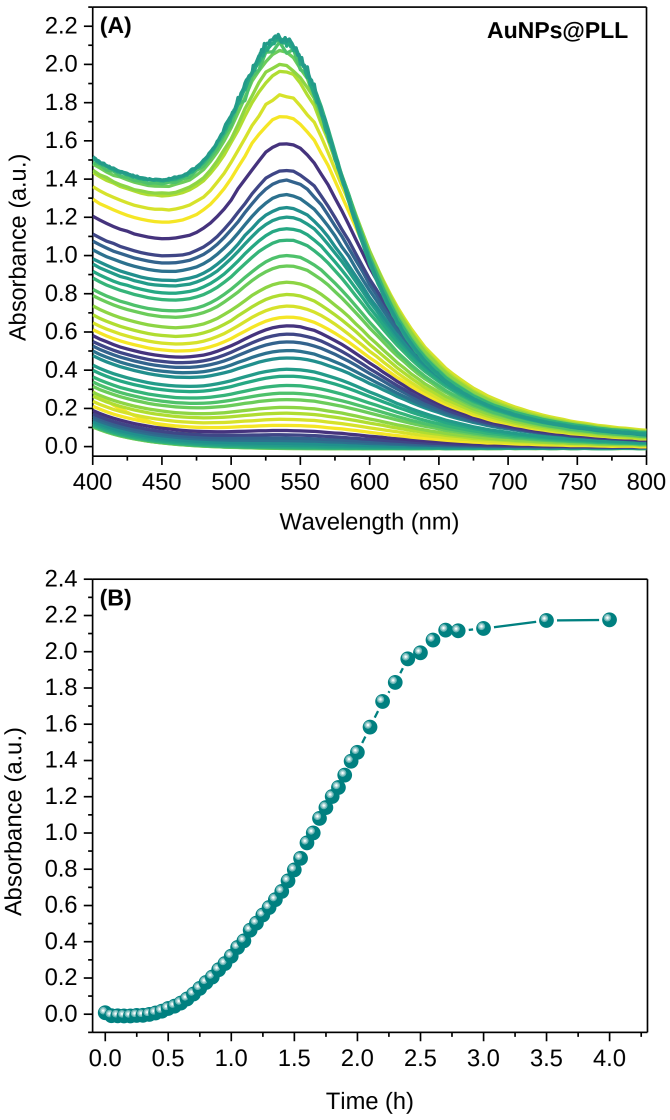

Figure S5: Evolution of UV-vis spectra for AuNPs@PLL in 10 mM PB at pH 7.4 at 75°C (A) and respective values of absorbance at λ_{max} nm as a function of time (B).

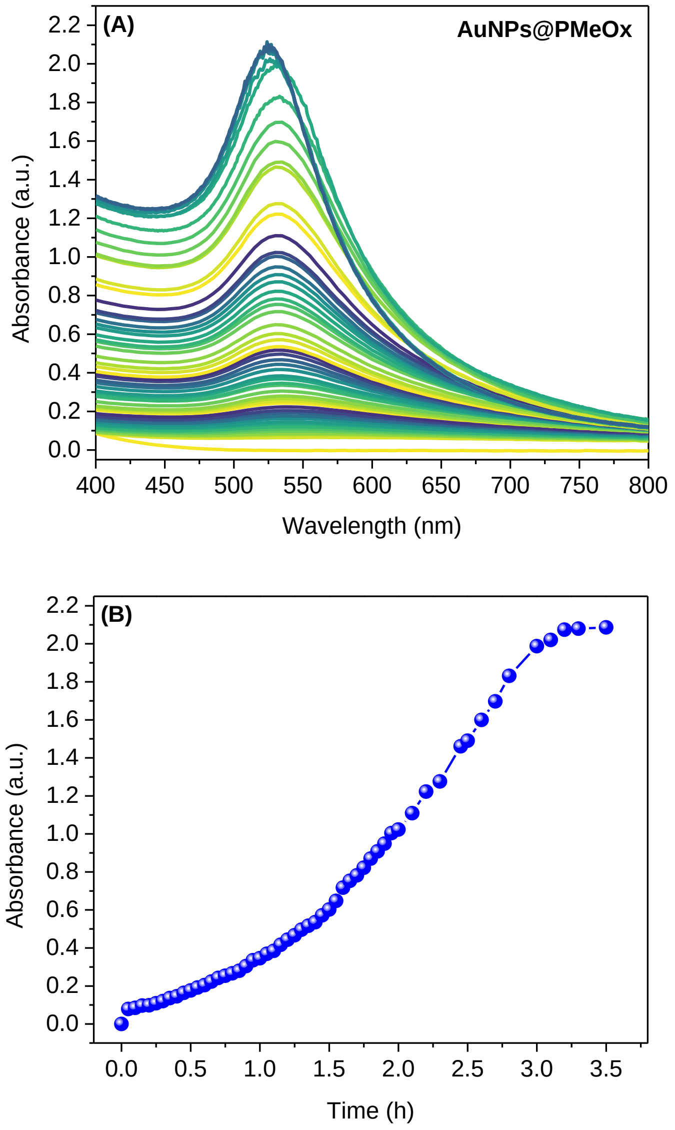

Figure S6: Evolution of UV-vis spectra for AuNPs@PMeOx in 10 mM PB at pH 7.4 at 75°C (A) and respective values of absorbance at λ_{max} nm as a function of time (B).

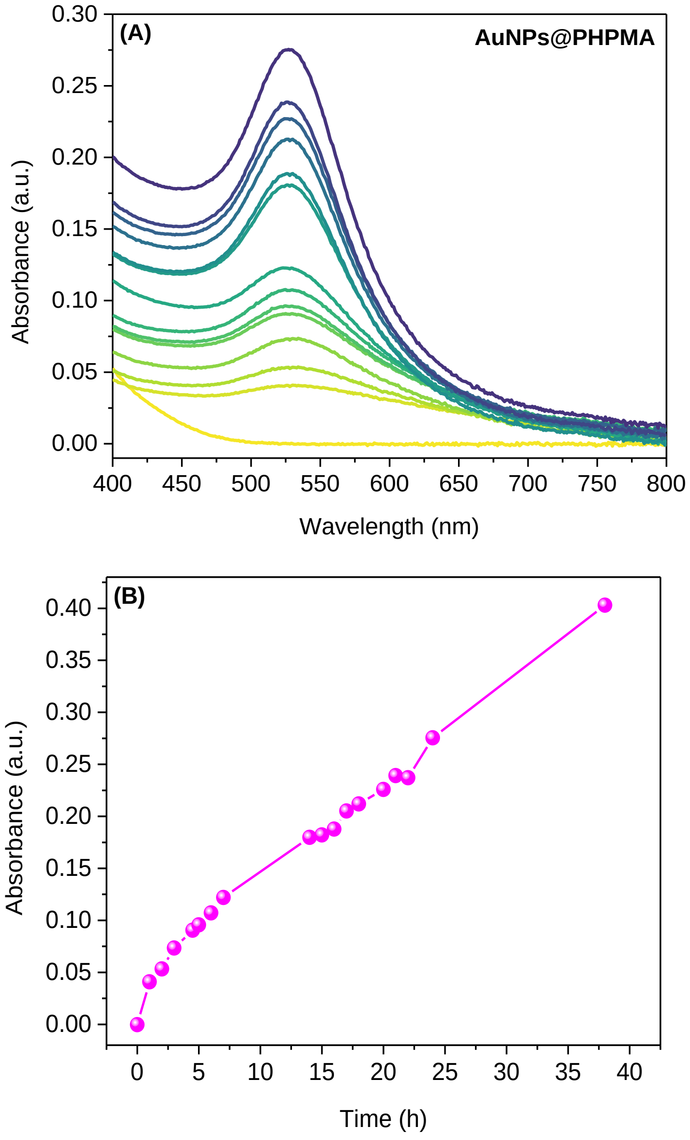

Figure S7: Evolution of UV-vis spectra for AuNPs@PHPMA in 10 mM PB at pH 7.4 at 75°C (A) and respective values of absorbance at λ_{max} nm as a function of time (B).

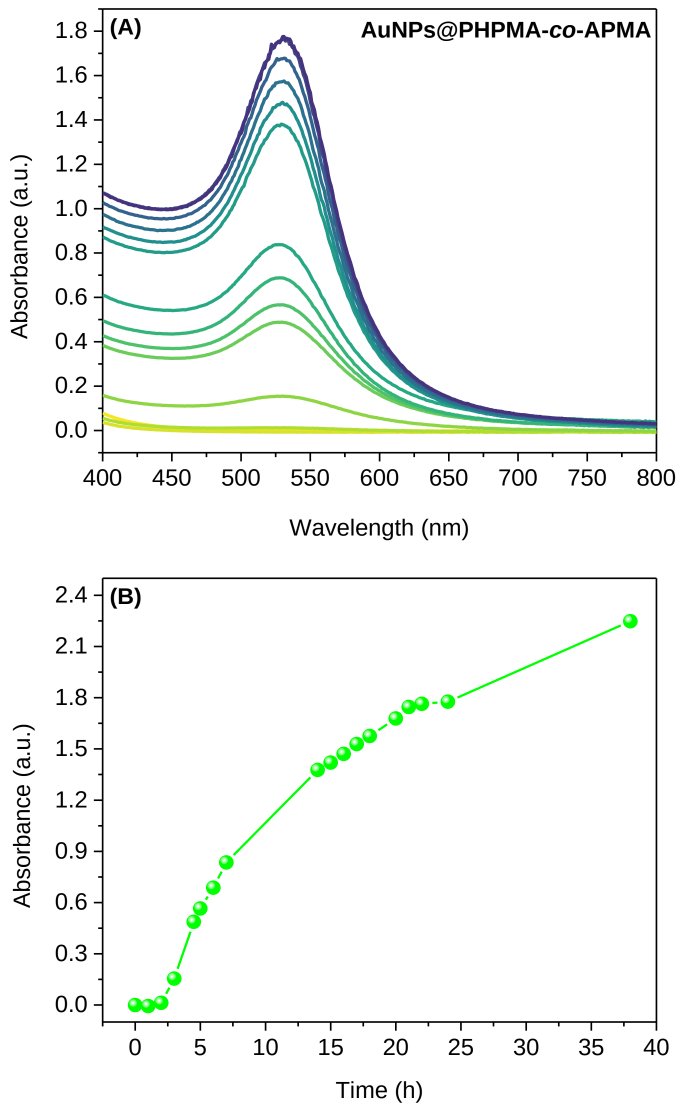

Figure S8: Evolution of UV-vis spectra for AuNPs@P(HPMA-co-APMA) in 10 mM PB at pH 7.4 at 75°C (A) and respective values of absorbance at λ_{max} nm as a function of time (B).

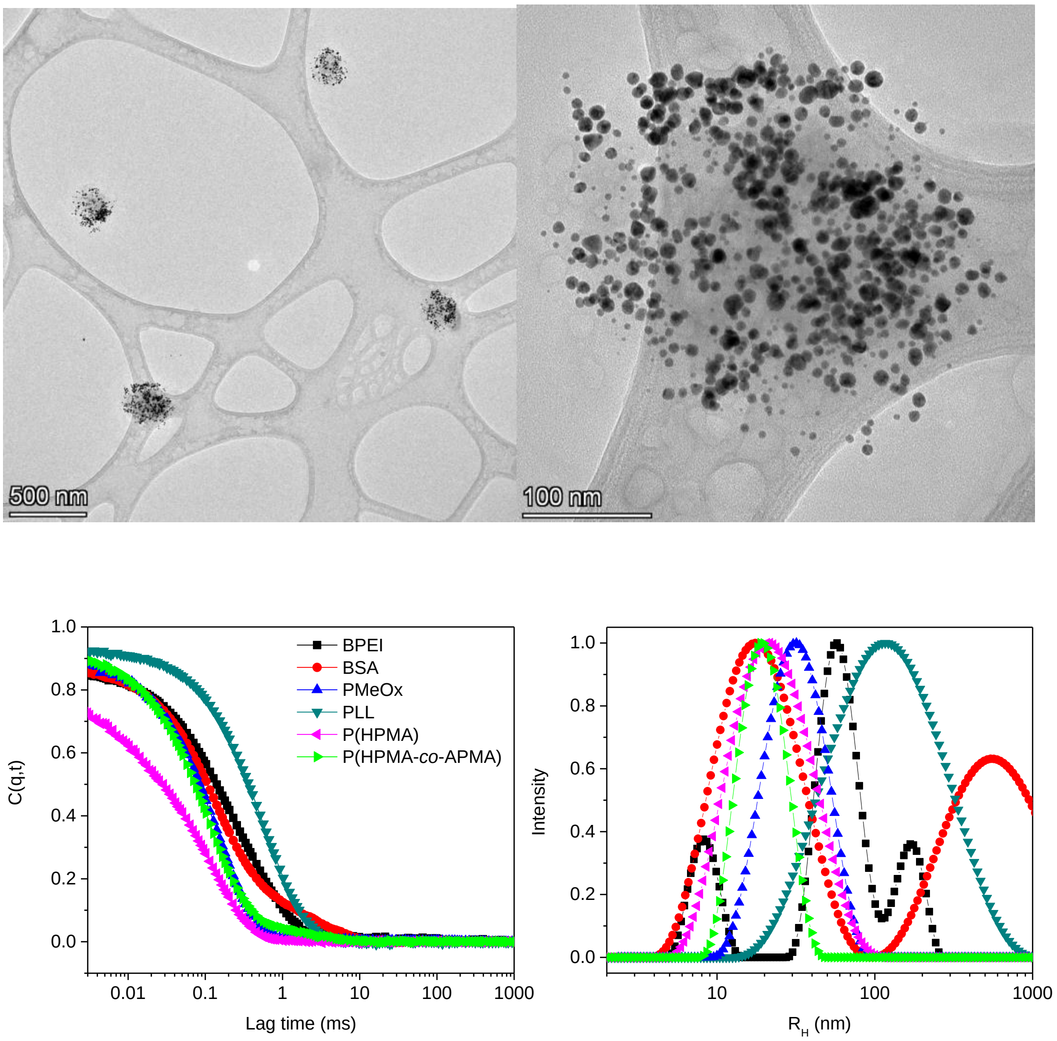

Figure S9. TEM micrographs at different magnifications showing aggregation of AuNPs@PLL particles in the dry state (top panels) and autocorrelation functions C(q,t) measured at 90° scattering angle (bottom left) with corresponding particle size distributions revealed by CONTIN analysis (bottom right) for all systems investigated in this study.

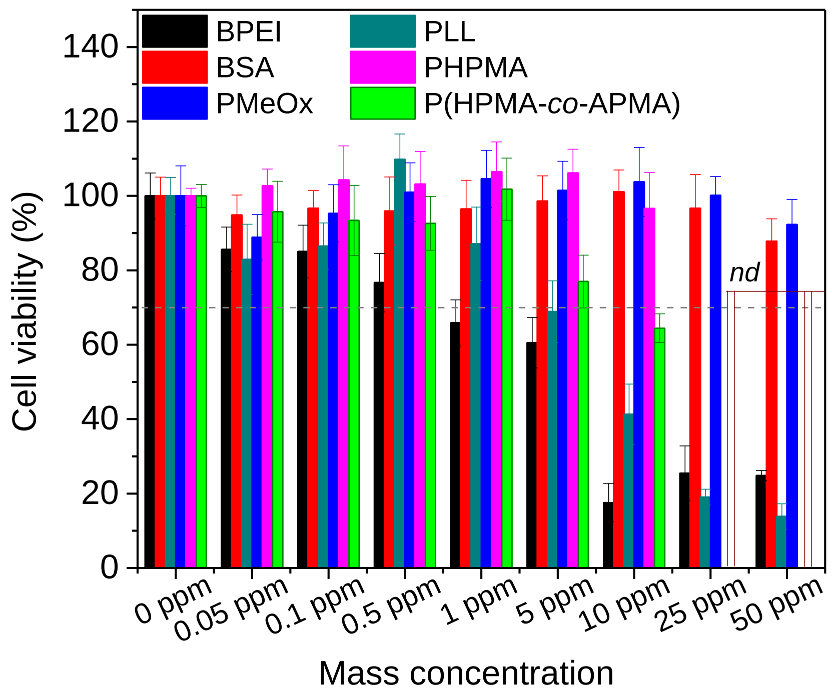

Figure S10: Dose-dependent viability of HeLa cells incubated with macromolecules of interest to this study, as indicated, represented in terms of mass concentration (ppm) of the respective AuNPs but in absence of them (*i.e.* polymer concentrations correspond to those present in AuNPs@macromolecules systems in Figure 6).

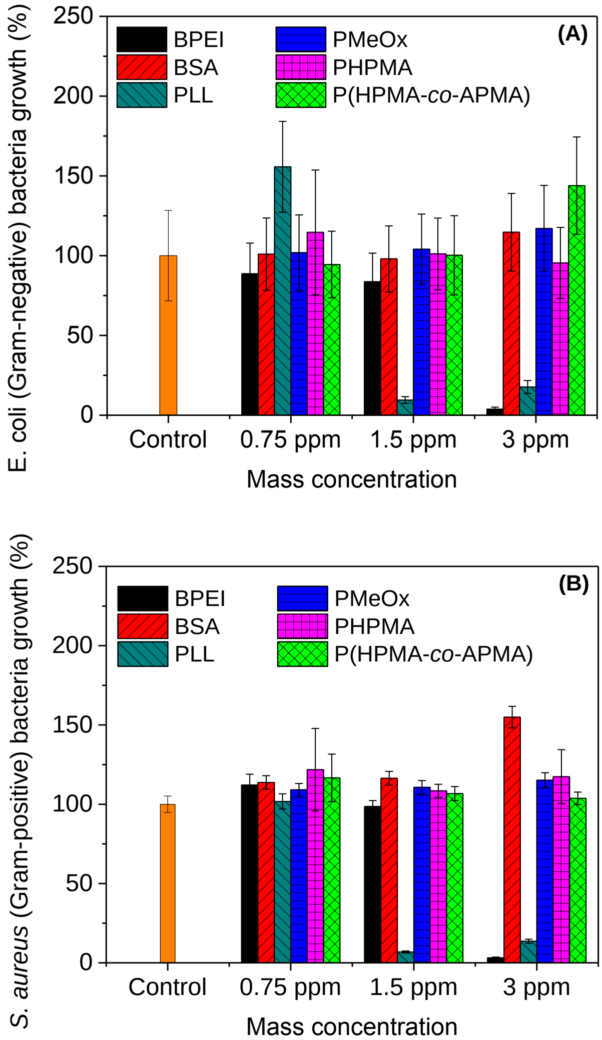

Figure S11. Bacterial growth of Gram-negative *E. coli* (A) and Gram-positive *S. aureus* (B) when in contact with distinct macromolecules at concentrations equivalent to amounts in AuNPs given in Figure 7 (manuscript) according to the labels.
